# Revealing the genetic basis of coat color in Tibetan sheep through selective sweep and transcriptomic analyses

**DOI:** 10.3389/fvets.2025.1711294

**Published:** 2025-11-24

**Authors:** Xue Li, Buying Han, Dehong Tian, Dehui Liu, Wulong Ma, Guangcai Bao, Lei Wang, Quanbang Pei, Zian Zhang, Kai Zhao

**Affiliations:** 1 Key Laboratory of Adaptation and Evolution of Plateau Biota, Qinghai Provincial Key Laboratory of Animal Ecological Genomics, Northwest Institute of Plateau Biology, Chinese Academy of Sciences, Xining, Qinghai, China; 2 University of Chinese Academy of Sciences, Beijing, China; 3 Menyuan County Agriculture, Animal Husbandry and Water Conservancy Comprehensive Service Center, Menyuan, Qinghai, China; 4 Qinghai Provincial Agricultural Project Guidance Service Center, Xining, Qinghai, China; 5 Qinghai Sheep Breeding and Promotion Service Center, Gangcha, Qinghai, China

**Keywords:** Tibetan sheep, coat color, whole genome sequencing, RNA-seq, molecular markers

## Abstract

**Background and objective:**

Coat color is an important economic trait in animal husbandry and plays a crucial role in the adaptability of animal. As a key economic trait in high-altitude areas, the genetic mechanisms regulating coat color in Tibetan sheep are not fully understood.

**Methods:**

In order to explore the genetic basis of coat color in Tibetan sheep, this study focused on white-coated Plateau Tibetan sheep (PT) and black-coated Guinan black fur sheep (GB). Using whole genome sequencing (10× coverage), transcriptomic analysis, Sanger sequencing, and protein structure prediction to identify candidate genes and molecular markers associated with coat color.

**Results:**

In this study, a total of 9.28 million SNPs were annotated from whole-genome sequencing. Compared with the white-coated PT sheep, the skin transcriptome of black-coated GB sheep exhibited 659 up-regulated and 426 down-regulated genes. *MC1R*, *MITF*, and *KIT* were identified as candidate genes for the coat color of Tibetan sheep. Through Sanger sequencing of all exons of these genes and association analysis with coat color phenotypes, we found that 8 of 12 SNPs were significantly associated with coat color in Tibetan sheep. The coat color of individuals with heterozygous or homozygous mutations in both SNPs (rs3508196008 and rs409651063) in the *MC1R* gene were black. These two SNPs were likely to not cause loss or alteration of protein function, but rather affected the coat color of Tibetan sheep by regulating gene transcription and expression levels, and can be applied as molecular markers to regulate coat color in Tibetan sheep.

**Conclusion:**

Two molecular markers (rs3508196008 and rs409651063) in the *MC1R* gene regulating Tibetan coat color were identified through selective sweep analysis, transcriptome, Sanger sequencing, and the genetic mechanism of coat color was analyzed, which provided new insights for the genetics of coat color in Tibetan sheep, and provided effective markers and technical support for molecular breeding.

## 1 Introduction

Coat color is an important phenotypic trait in animals, and play a key role in ecological adaptation, species evolution, and physiological functions, as well as breeding, health, and productivity (1). The genetic regulation of coat color involves not only the control of pigment synthesis, but also may be closely related to the physiological state, immune function, and environmental adaptation (2). With the advancement of animal breeding and genomics technology, coat color has become a key field in the study of animal genetics, particularly in understanding adaptation and species evolution (3).

The genetic mechanism of coat color have been a core topic in genetics and animal science research, and more than 400 genes that regulate animal coat color have been discovered (4). Classic pigment genes, such as Melanocortin 1 receptor (*MC1R*), Agouti signaling protein (*ASIP*), Tyrosinase-related protein 1 (*TYRP1*), Microphthalmia-associated transcription factor (*MITF*), and Kinase insert domain receptor (*KIT*), ultimately determine the coat color characteristics of animals by regulating the synthesis ratio of eumelanin (melanin) to pheomelanin (yellow pigment) in the skin and hair (5). As a key gene of the melanin production pathway, *MC1R* gene has been closely related to coat color variation in a variety of animals (6). With the advancement of genomics and high-throughput sequencing technology, researchers have discovered novel genes and non-coding regulatory regions related to coat color from a genome-wide perspective, further revealing the complexity of hair color regulation and the role of polygenic networks (7).

Despite significant advances in the study of coat color genetics in livestock, the genetic mechanisms underlying coat color remain poorly understood, particularly in high-altitude species like Tibetan sheep. This gap in knowledge, especially regarding the genetic regulation of coat color in these unique breeds, prompted us to conduct this study. Tibetan sheep, an important breed in high-altitude regions, exhibit distinct coat color phenotypes, with Plateau Tibetan sheep (PT) primarily white and Guinan black fur sheep (GB) having black fur. Understanding the genetic basis of these color variations is essential for both advancing basic genetic research and improving livestock breeding practices. Given the limited studies on the genetic mechanisms of coat color in Tibetan sheep, this research aims to fill this gap and provide valuable insights that could be applied in livestock breeding programs.

## 2 Materials and methods

### 2.1 Sample collection

The study involved two breeds of Tibetan sheep: 142 PT sheep from Gangcha County, Qinghai Province, China, characterized by a white coat covering 95% of their body surface, and 72 GB sheep from Guinan County, Qinghai Province, China, which have a black coat covering their entire body. Ear tip tissue samples were collected from each sheep and preserved in 95% alcohol. High-quality DNA was extracted using the TIANGEN^®^ TIANamp Genomic DNA Kit (Tiangen, Beijing, China). DNA purity and integrity were assessed by 1% agarose gel electrophoresis and spectrophotometer.

In accordance with relevant institutional Guinanlines and animal research ethics regulations, we euthanized three adult male individuals in the PT and GB groups. Sheep were deeply anesthetized by intravenous administration of 3% pentobarbital sodium (Solarbio, Beijing, China) and sacrificed by exsanguination at a healthy physiological stage. A 1 cm^2^ of skin tissue was collected from the same part of each sheep, and all samples were de-haired and adipose and connective tissue was stripped. Immediately after washing with phosphate buffer (PBS) to remove residual blood and contaminants, the sample is flash-frozen in liquid nitrogen for transcriptome analysis.

### 2.2 DNA extraction and whole-genome sequencing

A total of 20 adult individuals (half male and half female) were selected from both PT and GB Tibetan sheep breeds for whole-genome analysis. The PT sequencing data were obtained from our previous study (PRJNA1111723), while the GB samples were newly sequenced in this study. All samples were sequenced using the Illumina NovaSeq 6000 platform to target a depth of 10-fold coverage per genome.

### 2.3 Variant calling

The raw read quality was assessed using FastQC (v 0.21) with the default settings (8). High-quality paired-end reads (either 150 bp or 100 bp) were mapped to the sheep reference genome (ARS − UI_Ramb_v2.0^1^) by the Burrows–Wheeler Aligner (BWA v.0.7.12). The resulting SAM files were sorted and converted to BAM format using Picard software (v1.107). Since the library preparation steps may introduce duplicate reads, “MarkDuplicates” function of Picard’s was used to remove these duplicates, and only the reads with the highest mapping scores were retained for further analysis. The UnifiedGenotyper tool of the Genomic Analysis Toolkit (GATK v3.8) was used to detect SNPs and insertions and deletions (Indels) variants using strict quality control parameters (9). InDel variants were further filtered based on established quality criteria and missing sites were removed. Finally, ANNOVAR software was used to annotate the identified SNPs and InDels (10).

### 2.4 Population genetics analysis

Principal component analysis (PCA) was performed on the SNP data using GCTA software.^2^ Subsequently, a maximum likelihood phylogenetic tree was constructed using FastTree software.^3^ The reliability of the tree branches was assessed through bootstrapping with 1,000 replications.

Population structure analysis was performed using ADMIXTURE software^4^ based on SNP data. The admixture model was applied with a range of *K* values (2–6), representing the number of ancestral populations. Default settings were used for other parameters. The optimal *K* value, which best reflects the true underlying structure was determined based on cross-validation error.

### 2.5 Detection of selective signatures

We used the nucleotide diversity ratio (θπ _GB_/θπ _PT_) and population differentiation (*F*_ST_) values to identify genomic regions that are likely to have undergone strong selective sweeps. Chromosomes from the reference genome were selected for further analysis. Data analysis was performed using Vcftools (0.1.15) (11). Specifically, the *F*_ST_ value for each chromosome was calculated using a sliding window of 500 kb with a step size of 50 kb (12). Regions with extremely low or high θπ ratios (the lower and upper 5% tails) and significantly elevated *F*_ST_ values (the top 5% of *F*_ST_ values) were selected as regions potentially affected by strong selective sweeps.

### 2.6 Gene annotation, GO, and KEGG analyses

Genes within the selected regions were annotated based on sheep reference assembly ARS-UI_Ramb_v2.0. Functional enrichment analysis was conducted using the Gene ontology (GO) database^5^ and the Kyoto encyclopedia of genes and genomes (KEGG) database.^6^ We identified GO terms and KEGG pathways with significant enrichment at *p* value threshold of 0.05.

### 2.7 RNA-seq analysis

RNA extraction, sequencing, and transcriptome profiling were conducted on three biological replicates of skin samples from the same shoulder region of GB and PT sheep. Total RNA was extracted from each sample using TRIzol (Invitrogen, Carlsbad, CA, United States), followed by assessments of degradation and contamination on 1% agarose gels and verification of purity using a Nanodrop® spectrophotometer (Thermo, Carlsbad, CA, United States). RNA concentration was quantified using a Qubit® RNA Assay Kit and Qubit^®^ 2.0 Fluorometer (Life Technologies, Carlsbad, CA, United States). The RNA integrity was evaluated using an RNA Nano 6000 Assay Kit on an Agilent Bioanalyzer 2100 system (Agilent Technologies, Santa Clara, CA, United States). Each sample utilized 1.5 μg of RNA as input material for RNA sample preparation. Subsequently, sequencing libraries were produced with the MGIEasy RNA Library Prep Kit (BGI Co., Ltd., Shenzhen, Guangdong, China) according to the manufacturer’s instructions. The resulting library was sequenced on the DNBSEQ-T7 platform.

SAMtools filtering was employed for quality control of the read data (13). A reference genome index was subsequently constructed, and high-quality RNA-seq reads were subsequently aligned to the reference genome using HISAT2 (v2.0.4) (14). Differential expression analysis was carried out using the R package DESeq2 (v1.38.3) based on read count numbers (15). The statistical significance of variations in gene expression was assessed through the Wald test, with a threshold of *p* < 0.05 deemed significant. Enrichment analyses of GO and KEGG pathways.

Differential alternative splicing (AS) analysis of RNA-Seq data was performed using the rMATS software.^7^ The exon inclusion level (ф) was used to quantify alternative splicing, representing the percentage of transcripts containing the alternative splicing event region in relation to those with the skipped region. The △ф (△ф = |ф1 - ф2|) and false discovery rate (FDR) values were used to confirm whether there were differential alternative splicing events between the two sample groups. ф1 and ф2 represent the exon inclusion levels for the two groups of samples. When △ф > 5% and FDR ≤ 1%, differential alternative splicing at that splice site is considered to have occurred between the two groups.

### 2.8 Transcriptome validation

cDNA synthesis was performed on the remaining RNA from transcriptome sequencing using a reverse transcription kit (PrimeScript™ 1st Stand cDNA Synthesis Kit). Specific primers for the selected genes were designed using Primer 5 software, with the sheep Glyceraldehyde-3-phosphate dehydrogenase (*GAPDH*) gene serving as the internal reference gene (Supplementary Table S1). For each target gene and the internal reference gene, the cDNA template from the sample was used for PCR reaction. The PCR reaction system is detailed in Supplementary Table S2. The PCR reaction mixture was then subjected to amplification on a real-time PCR instrument. The cycling conditions were as follows: pre-denaturation at 95 °C for 30 s; 40 cycles of denaturation at 95 °C for 15 s, and annealing at 60 °C for 30 s; followed by a total of melting curve analysis from 65 °C to 95 °C, with fluorescence signals collected every 0.5 °C increment. Each RT-qPCR reaction was performed in triplicate. Relative gene expression levels were calculated using the 2^−ΔΔ^CT method. Finally, the Pearson correlation coefficient was calculated to assess the relationship between the transcriptome data and the RT-qPCR gene expression results.

### 2.9 SNP identification and sequencing

This study identified candidate genes related to Tibetan sheep coat color through selective sweep analysis and transcriptome analysis. Primers for all exons of the candidate genes were designed using Primer3 v0.4.0, and the primer sequence information is provided in Supplementary Table S3. Subsequently, Sanger sequencing was performed on 142 white PT sheep and black GB sheep to identify SNPs regulating the coat color of Tibetan sheep. PCR amplification was then performed in a 30 μL reaction volume containing 1.0 μL of DNA, 15 μL of 2 × Taq PCR Master Mix, 1.0 μL of each primer, and double-distilled water (ddH_2_O) to reach the final volume. Amplification was carried out using a Bio-Rad S1000 thermal cycler (Bio-Rad, Hercules, CA, United States) with the following program: initial denaturation at 94 °C for 2 min, 35 cycles of denaturation at 94 °C for 10 s, annealing at 60 °C for 30 s, and elongation at 72 °C for 60 s, PCR products were then visualized on 1.0% agarose gels to assess the quality and quantity of the amplicons. Mutations were identified by Sanger sequencing using the Agilent 3730 system (Santa Clara, CA, United States). Sequence analysis was performed with DNAMAN version 5.2.10 (Lynnon BioSoft, Vaudreuil, Canada).

Linkage disequilibrium (LD) analysis of the candidate SNPs was conducted using Haploview v4.2 (Broad Institute, Cambridge, MA, United States) with default parameters under the confidence-interval algorithm. A chi-square test was performed using SPSS software (version 18.0, SPSS Inc., Chicago, IL, United States) to evaluate the association between the identified SNPs and the coat color phenotype in Tibetan sheep. SNPs with linkage disequilibrium (*r*^2^ > 0.8) and nominal significance (*p* < 0.05) were initially considered associated with coat color. To minimize false positives resulting from multiple comparisons, *p*-values were adjusted using the Bonferroni correction, and SNPs with corrected *p* < 0.0042 were regarded as significantly associated with coat color.

### 2.10 Protein structure analysis

The online tool ESPript^8^ was used to predict the secondary structures and identify structural elements in protein sequences. The three-dimensional (3D) structure of the protein was obtained from the Swiss-Model database^9^ and subsequently visualized.

## 3 Results

### 3.1 Whole-genome sequencing and variation

In this study, whole genome sequencing data of 40 Tibetan sheep (20 PT sheep and 20 GB sheep) were analyzed. The quality evaluation showed that the sequencing data had no fuzzy bases, the average GC content was 45.14%, the average base recognition accuracy of Q20 sequencing was 97.17%, and the accuracy of Q30 sequencing was 92.72% (Supplementary Table S4). These indicators all met the established standards of Q20 value needed to be more than 90%, Q30 value had to be higher than 85%, and GC content was close to 50%. This confirmed that the sequencing data has high quality characteristics.

To ensure the quality of subsequent data analysis, the raw sequencing data must be deeply filtered to obtain high-quality results. After screening, an average of 182.66 million high-quality reads and 27.13 billion base pairs were obtained per sample (Supplementary Table S5). The average coverage of the sequencing genome reached 99.82%, ranging from 99.64 to 99.91% (Supplementary Table S6). Therefore, the sequencing quality and the chosen reference genome met the requirements for further analyses.

### 3.2 Characterization of the variants

In this study, we annotated a total of 9.28 million SNPs across 20 categories. The majority of these SNPs were located in intergenic regions (59.25%), followed by intronic regions (37.43%) and exonic regions (0.87%) (Supplementary Table S7). Additionally, we identified and annotated 4.08 million Indels across 24 categories. Most of these Indels were found in intergenic regions (60.24%), followed by introns (36.88%) and 3′ untranslated regions (3′UTRs) (0.88%) (Supplementary Table S8).

### 3.3 Phylogenetic relationships and population structure

**Figure 1:**
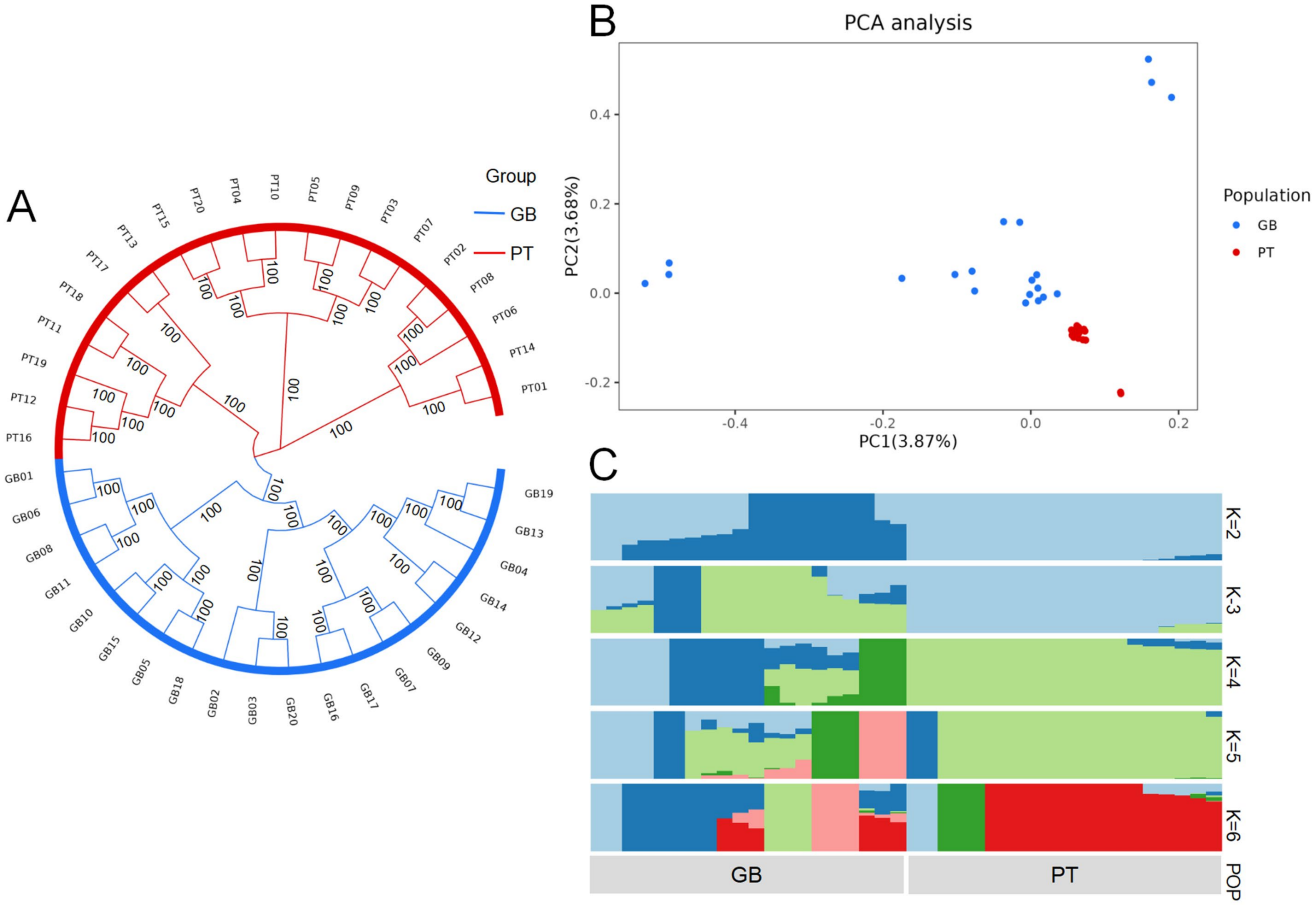
Phylogenetic relationships and population structure of Plateau Tibetan sheep and Guinan black fur sheep. GB, Guinan Black Fur sheep; PT, Plateau Tibetan sheep. **(A)** A phylogenetic tree of Plateau Tibetan sheep and Guinan black fur sheep using the maximum likelihood algorithm in fast tree software. **(B)** PCA of Plateau Tibetan sheep and Guinan black fur sheep. **(C)** The population genetic structure of Plateau Tibetan sheep and Guinan black fur sheep. Each column in the image represents an individual, and the length of each different colored segment indicates the proportion of an ancestor in the individual’s genome. The labels on the image denote the groups. When *K* = 2, the inferred structure best represents the underlying population structure.

Analysis of the genetic structure of the populations of the two Tibetan sheep revealed significant separations in both phylogenetic trees and PCA (Figures 1A,B). This distinct separation confirmed that the selected samples were valid and accurately represented the two genetically differentiated populations.

The ADMIXTURE software was used to assess the proportions of shared ancestry between the two breeds. An unsupervised clustering model was applied to investigate population structures across a range of ancestral groups (*K* = 2–6). Genetic structure analysis showed that when *K* = 2, the two Tibetan sheep breeds were derived from different ancestral lineages. Most of the genetic makeup of the GB breed derived from one ancestral group (dark blue), whereas the PT breed showed predominant genetic contributions from a different ancestral group (light blue) (Figure 1C).

### 3.4 Detection of selective signatures

**Figure 2:**
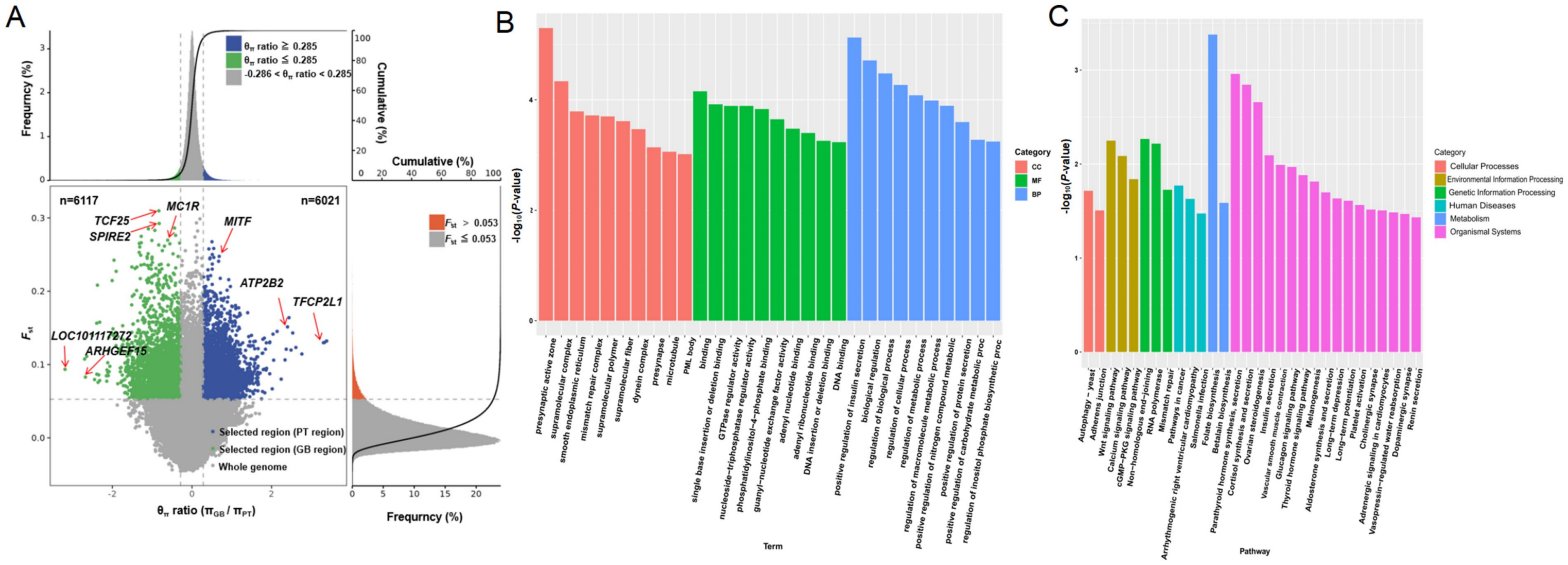
Selective sweep analysis of Plateau Tibetan sheep and Guinan black fur sheep. GB, Guinan Black Fur sheep; PT, Plateau Tibetan sheep. **(A)** The scatter plot of genome-wide selective sweep signals. The horizontal axis represents the ratio of the θπ _sample_/θπ _control_, the vertical axis represents the *F*_ST_ value. The blue area represents the selected area for Plateau Tibetan sheep, while the green area represents the selected area for Guinan Black Fur sheep. **(B)** KEGG enrichment analysis of selectively swept genes. **(C)** GO enrichment analysis of selectively swept genes.

In order to identify genomic regions with strong selection pressure in two different coat color regions of Tibetan sheep, two analysis methods were used: θπ GB/θπ PT ratio distribution and *F*_ST_ value analysis (Figure 2A; Supplementary Table S9). When the θπ ratio was ≤ −0.286 and the *F*_ST_ value was >0.053, it indicated that there was strong selection pressure in the black hair GB sheep, involving a total of 6,117 regions. On the contrary, when the θπ ratio was ≥0.285 and the *F*_ST_ value was ≥0.053, strong selection pressure was detected in the white-haired PT sheep, with a total of 6,021 regions identified. Notably, genes related to melanin production, including *MC1R* and *MITF*; genes involved in metabolic processes, such as transcription factor CP2-like protein 1 isoform X1 (*TFCP2L1*) and plasma membrane calcium-transporting ATPase 2 (*ATP2B2*); genes associated with the nervous system, angiogenesis, and DNA damage repair, such as Rho guanine nucleotide exchange factor 15 (*ARHGEF15*) and protein spire homolog 2 (*SPIRE2*); as well as immune-related genes like *LOC101117272*, were subject to strong selective pressures.

These genes were not only enriched in the core pigment-related pathway of melanogenesis but were also highly active in pathways associated with environmental adaptation and stress response, including the thyroid hormone signaling pathway, vasopressin-regulated water reabsorption, aldosterone synthesis and secretion, calcium signaling pathway, and cGMP-PKG signaling pathway. Additionally, they participated in pathways related to muscle function and cardiovascular regulation, such as vascular smooth muscle contraction and adrenergic signaling in cardiovascular cells. Furthermore, these genes were involved in metabolism and hormone regulation pathways, including ovarian steroidogenesis, insulin secretion, glucagon signaling, and renin secretion. They also contributed to DNA damage repair and genome stability pathways, such as non-homologous end joining, mismatch repair, and adherens junction, as well as signal transduction pathways, including the dopaminergic synapse, Wnt signaling pathway, RNA polymerase, and axon guidance (Figures 2B,C; Supplementary Tables S10, S11).

### 3.5 RNA-seq analysis

**Figure 3:**
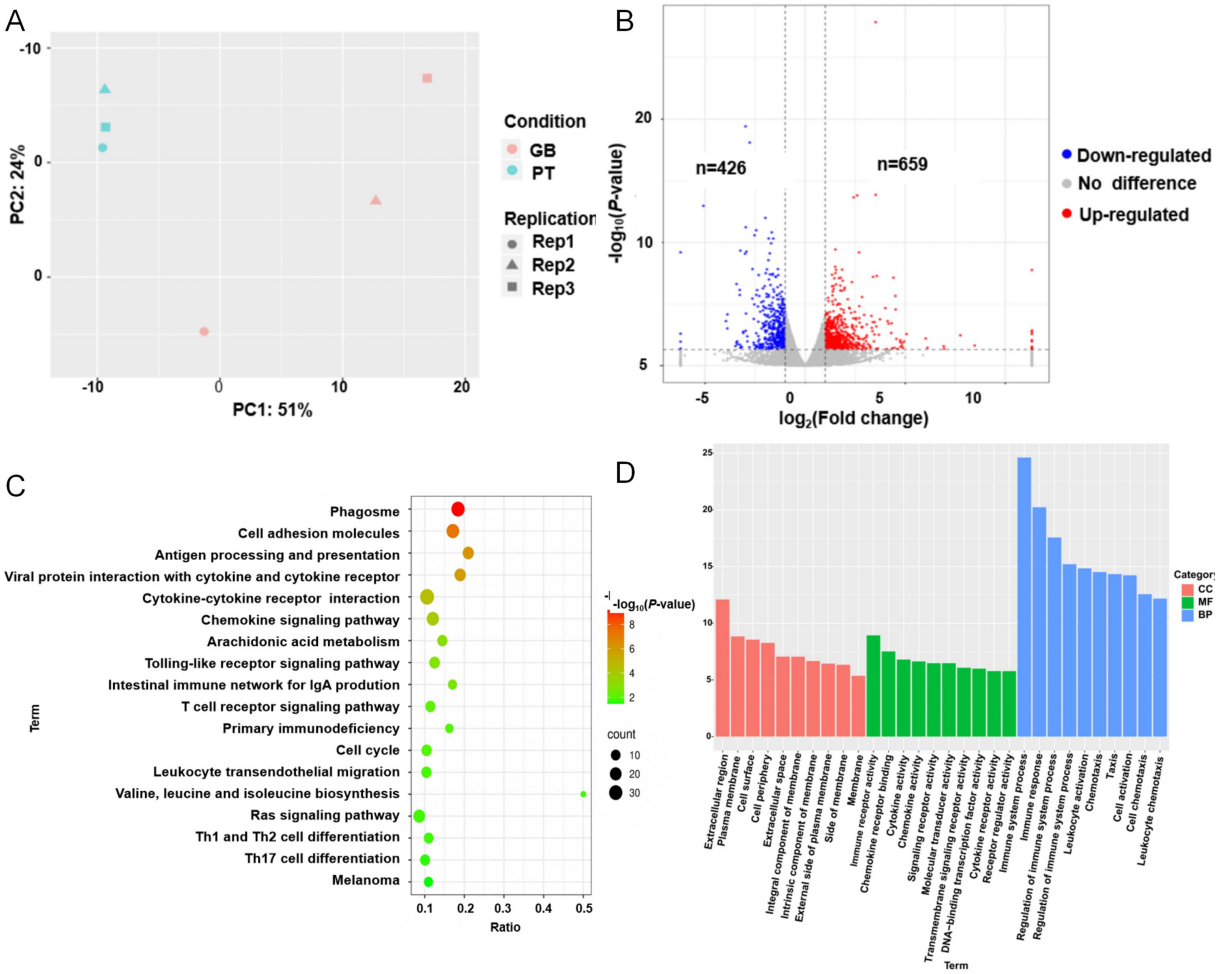
Skin transcriptome results of Plateau Tibetan sheep and Guinan black fur sheep. GB, Guinan Black Fur sheep; PT, Plateau Tibetan sheep. **(A)** PCA diagram of the skin transcriptome of Plateau Tibetan sheep and Guinan black fur sheep. **(B)** Volcano map of DEGs. Red represents upregulated DEGs, blue represents downregulated DEGs. **(C)** Bubble map of enriched KEGG pathways. **(D)** Bar graphs of enriched GO pathways.

To investigate the key genes and pathways involved in coat color formation, transcriptome sequencing analysis was performed on skin tissues from black GB sheep and white PT sheep. The average data yield per sample was 7.80 Gb, with 96.28% of bases reaching Q30, and an average of 7.63 Gb of clean data per sample. The mean alignment rate to the reference genome was 98.66% (Supplementary Tables S12, S13). PCA demonstrated a clear separation between the two groups (Figure 3A), indicating that the transcriptome data were of sufficient quality for subsequent analyses.

Comparative analysis revealed 659 upregulated and 426 downregulated genes in black GB sheep relative to white PT sheep (Figure 3B). Functional enrichment analysis indicated that these differentially expressed genes (DEGs) were mainly associated with four categories of pathways: pigmentation, immunity and inflammation, signal transduction and cellular regulation, and metabolism and nutrition. Pigmentation-related pathways, including melanogenesis and melanom. While immune and inflammation pathways included phagosome, Toll-like receptor signaling, natural killer cell–mediated cytotoxicity, T cell receptor signaling, Th1/Th2/Th17 cell differentiation, and cytokine–cytokine receptor interaction were also significantly enriched. Signal transduction and cellular regulation pathways included the Rap1 and Ras signaling pathway, regulation of the actin cytoskeleton, cell cycle, and osteoclast differentiation were enriched. Metabolism and nutrition-related pathways included arachidonic acid metabolism, valine/leucine/isoleucine biosynthesis, glutathione metabolism, and mineral absorption, were also significantly enriched (Figures 3C,D; Supplementary Tables S14, S15).

In this study, six genes were upregulated in the melanin pathway: *KIT*, *TYRP1*, Tyrosinase (*TYR*), *MC1R*, Wnt family member 4 (*WNT4*), and Phospholipase C beta 2 (*PLCB2*). Conversely, three genes—Frizzled class receptor 2 (*FZD2*), Lymphoid enhancer binding factor 1 (*LEF1*), and Frizzled class receptor 3 (*FZD3*) were downregulated (Supplementary Table S16). AS analysis identified 6,300 variable 3′ splice sites (A3SS), 7,145 variable 5′ splice sites (A5SS), 7,269 mutually exclusive exons (MXEs), 2,210 retained introns (RIs), and 42,785 skipping exons (SE) across all genes (Supplementary Figure S1). Notably, no significant AS events were observed for DEGs in the melanogenesis pathway, suggesting that differential expression rather than AS may primarily contribute to coat color variation in these samples.

### 3.6 Transcriptome validation

To ensure the accuracy and reliability of transcriptome data, we selected eight DEGs for qPCR validation, which were either significantly differentially expressed or associated with melanin production. As shown in Supplementary Table S17, the correlation coefficient (*r*^2^) between qPCR results and transcriptome data reached 0.92, which was significantly higher than the threshold of 0.8, with a *p* value < 0.0001. These results confirmed that the transcriptome data were reliable and suitable for subsequent analyses.

### 3.7 SNP identification

Selective sweep and transcriptome analysis revealed that the melanogenesis pathway, directly related to skin pigmentation and melanocyte activity, was significantly enriched. In this pathway, *MITF*, *KIT*, and *MC1R* were identified as having strong selective pressure and differential expression. Therefore, these genes were selected as candidate genes for Tibetan coat color. Primers were designed for all exons of each gene, and Sanger sequencing was performed to identify SNPs potentially associated with coat color.

**Figure 4:**
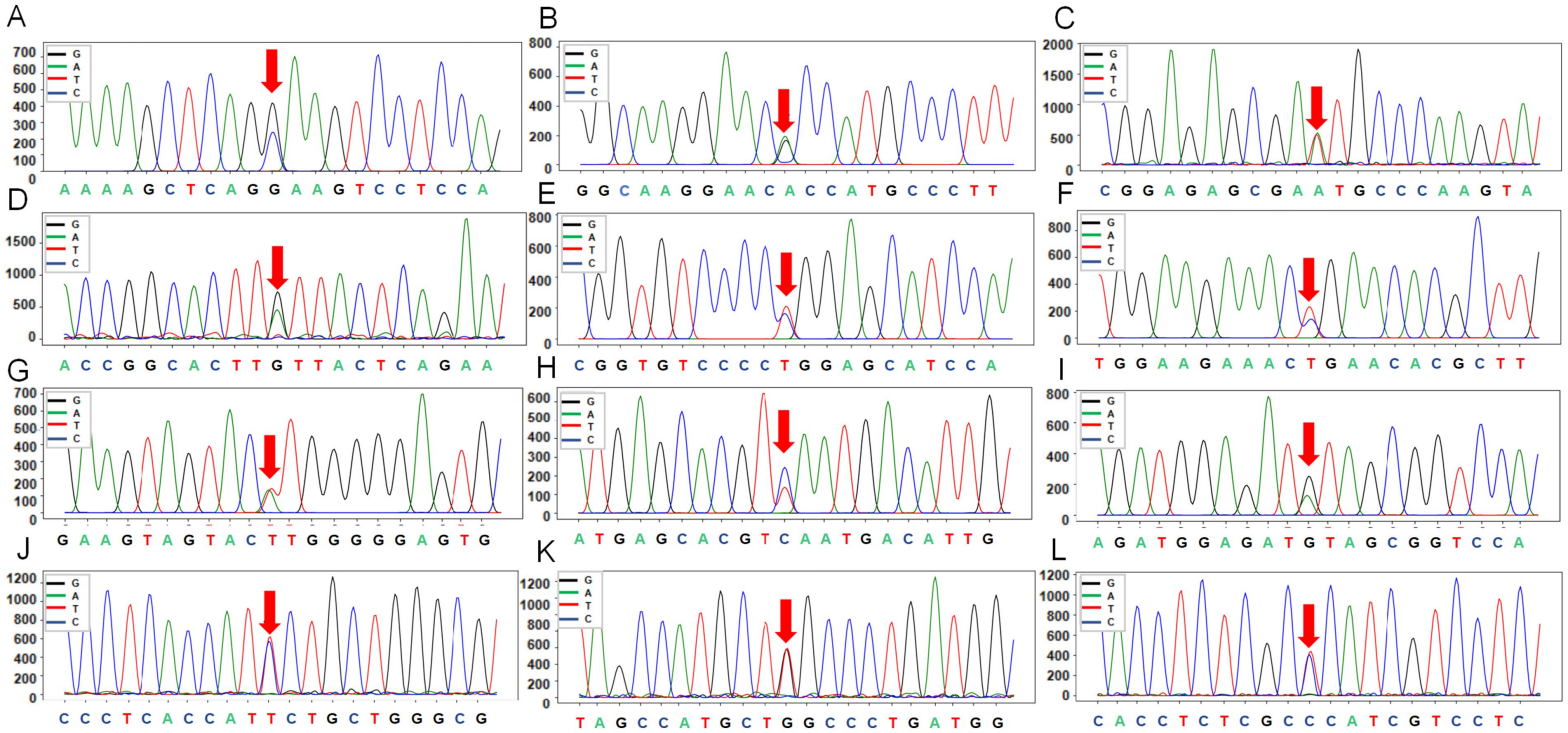
The peak maps of 12 SNPs from three genes (*KIT*, *MITF*, *MC1R*) in Plateau Tibetan sheep and Guinan Black Fur sheep. **(A)** *KIT* mutations marked by the red arrows are located in exon 15 of *KIT* (rs404963453), with reverse sequencing. **(B)** *KIT* mutations marked by the red arrows are located in exon 15 of *KIT* (rs416169878), with reverse sequencing. **(C)** *MITF* mutations marked by the red arrows are located in exon 4 of *MITF* (g.31615279302), with reverse sequencing. **(D)** *MITF* mutations marked by the red arrows are located in exon 9 of *MITF* (rs161248978), with reverse sequencing. **(E)** *MITF* mutations marked by the red arrows are located in exon 10 of *MITF* (rs161248955), with reverse sequencing. **(F)** *MITF* mutations marked by the red arrows are located in exon 10 of *MITF* (rs416988818), with forward sequencing. **(G)** *MC1R* mutations marked by the red arrows are located in exon 1 of *MC1R* (rs3508196008), with forward sequencing. **(H)** *MC1R* mutations marked by the red arrows are located in exon 1 of *MC1R* (rs409651063), with forward sequencing. **(I)** *MC1R* mutations marked by the red arrows are located in exon 1 of *MC1R* (rs160910030), with forward sequencing. **(J)** *MC1R* mutations marked by the red arrows are located in exon 1 of *MC1R* (rs398814350), with forward sequencing. **(K)** *MC1R* mutations marked by the red arrows are located in exon 1 of *MC1R* (rs412064209), with forward sequencing. **(L)** *MC1R* mutations marked by the red arrows are located in exon 1 of *MC1R* (rs596064420), with forward sequencing.

A total of 12 SNPs were identified in the exon regions of the three genes (Figure 4; Supplementary Table S18). In the *KIT* gene, two SNPs were detected in exon 15: rs404963453 (a synonymous) and rs416169878 (a nonsynonymous, resulting in an alanine-to-valine substitution at position 784). In the *MITF* gene, four synonymous SNPs were identified: g.31615279302(A > T) in exon 4, rs161248978 in exon 9, and rs161248955 and rs416988818 in exon 10. In the *MC1R* gene, six SNPs were found in exon 1, of which three were synonymous (rs160910030, rs398814350, rs412064209). The remaining three were nonsynonymous, resulting in amino acid substitutions: methionine at position 73 was replaced by lysine (rs3508196008), aspartate at position 121 was replaced by asparagine (rs409651063), and glutamate at position 263 was replaced by proline (rs596064420).

**Figure 5:**
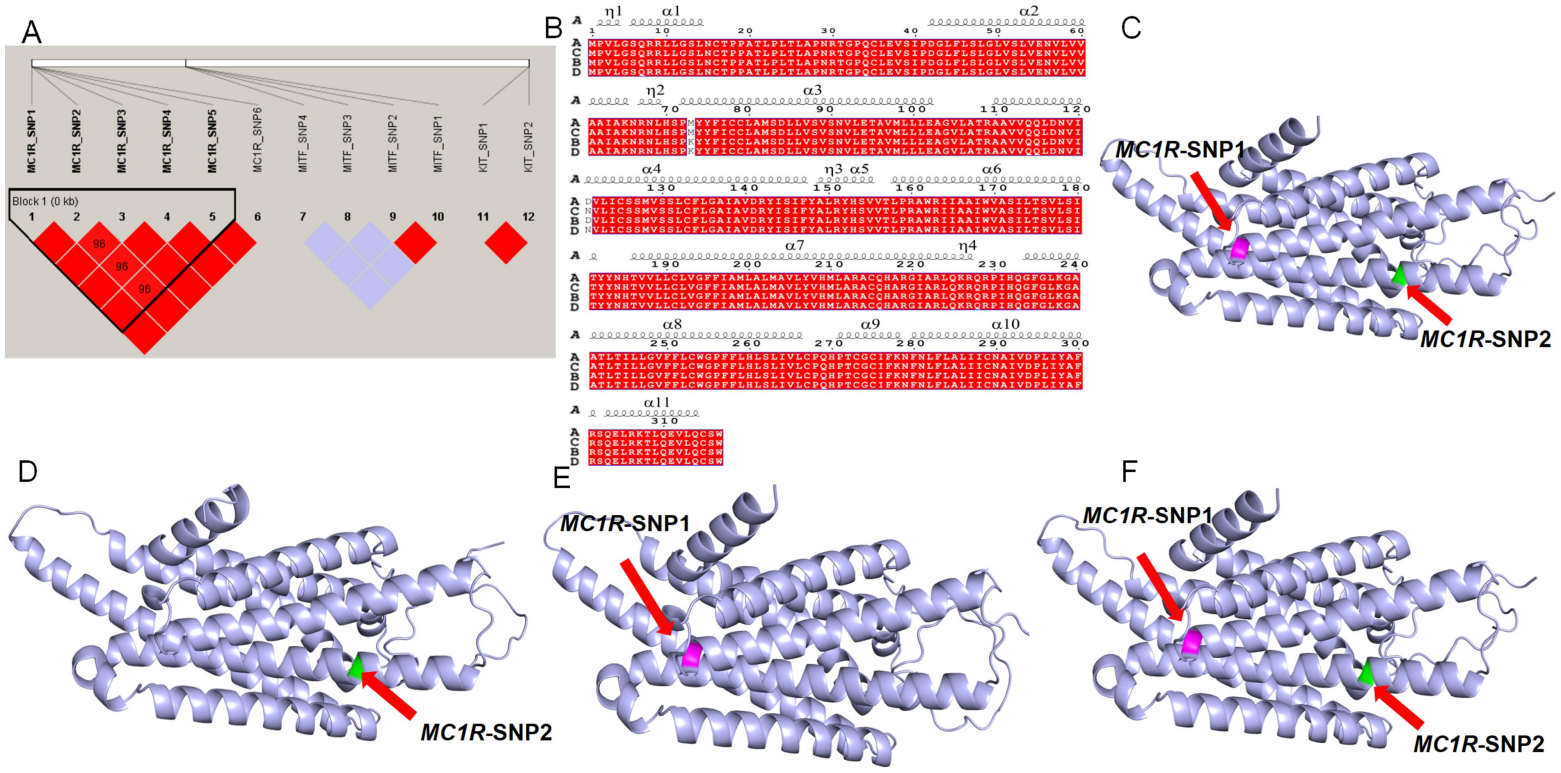
Linkage disequilibrium analysis and protein structure prediction. **(A)** Linkage disequilibrium analysis of 12 SNPs. Red regions indicate SNPs in linkage disequilibrium within that region (D′ > 0.85). **(B)** Protein secondary structure prediction. A: wild-type protein sequence; B: *MC1R*-SNP1 protein sequence; C: *MC1R*-SNP2 protein sequence; D: protein sequence carrying both *MC1R*-SNP1 and *MC1R*-SNP2. α denotes and *η* denotes 3₁₀ helices. **(C)** Three-dimensional structure of the wild-type protein; the purple region indicates the amino acid at position 73 affected by the *MC1R*-SNP1 mutation, and the green region indicates the amino acid at position 121 affected by the *MC1R*-SNP2 mutation. **(D)** Three-dimensional structure of the *MC1R*-SNP1 protein; **(E)** three-dimensional structure of the *MC1R*-SNP2 protein; **(F)** three-dimensional structure of the protein carrying both *MC1R*-SNP1 and *MC1R*-SNP2.

LD analysis showed strong linkage among SNP1–SNP6 of *MC1R* (*r*^2^ = 0.96), as well as between SNP1 and SNP2 of *MITF* and *KIT* (Figure 5A). Association analysis between these SNPs and coat color indicated that eight SNPs—*KIT*-SNP2, *MITF*-SNP3, *MITF*-SNP4, *MC1R*-SNP1, *MC1R*-SNP2, *MC1R*-SNP3, *MC1R*-SNP4, and *MC1R*-SNP5—were significantly associated with coat color (*r*^2^ > 0.8, *p* < 0.01; Table 1). Notably, the presence of allele A at *MC1R*-SNP1 (T/A) or *SNP2* (G/A) was consistently associated with a black coat phenotype, suggesting these two SNPs could serve as potential molecular markers for coat color in Tibetan sheep.

**Table 1:** Association analysis of SNPs and coat color in Tibetan sheep.

SNP	Genotype	GB	PT	χ^2^	*P*-value	Significance after correction
*KIT*-SNP1	CC	2	3	5.540	0.063	ns
	CG	15	52			
	GG	55	87			
*KIT*-SNP2	AA	7	0	30.836	<0.0001	*
	GA	30	26			
	GG	35	115			
*MITF*-SNP1	AT	0	3	1.543	0.552	ns
	AA	72	139			
*MITF*-SNP2	GA	0	5	2.560	0.172	ns
	GG	71	137			
*MITF*-SNP3	CT	7	23	13.724	<0.0001	*
	CC	64	119			
*MITF*-SNP4	TT	0	32	49.240	<0.0001	*
	CT	19	74			
	CC	52	36			
*MC1R*-SNP1	AA	29	0	212.000	<0.0001	*
	AT	43	0			
	TT	0	140			
*MC1R*-SNP2	TT	31	0	212.000	<0.0001	*
	CT	41	0			
	CC	0	140			
*MC1R*-SNP3	GA	13	69	50.651	<0.0001	*
	AA	59	45			
	GG	0	28			
*MC1R*-SNP4	GT	13	69	50.651	<0.0001	*
	GG	59	45			
	TT	0	28			
*MC1R*-SNP5	CT	13	69	50.651	<0.0001	*
	TT	59	45			
	CC	0	28			
*MC1R*-SNP6	CT	7	26	2.766	0.112	ns
	TT	65	115			

GB, Guinan Black Fur sheep; PT, Plateau Tibetan sheep. The table lists the genotype of each SNP, the number of individuals with the genotype in GB and PT, as well as the chi-square value (χ^2^) and the significance level (*P*-value). ns, Not significant. ^*^Significant after Bonferroni correction (adjusted *P* < 0.0042).

### 3.8 Protein structure analysis

LD and association analyses with coat color phenotypes revealed a strong linkage between *MC1R*-SNP1 and *MC1R*-SNP2, with the SNP1 mutation frequently co-occurring with the SNP2 mutation. Therefore, protein structure predictions were performed for proteins carrying SNP1, SNP2, or both mutations. Compared with the wild-type sequence, the predicted secondary structures (Figure 5B) and tertiary 3D structures (Figures 5C–F) of the mutated proteins showed overall similarity, containing 11 α-helices (α1-α11) and 3₁₀-helices (η1-η4), with no substantial deviations observed relative to the wild-type protein.

## 4 Discussion

Coat color is not only an important feature of animal appearance, but also closely related to environmental adaptability, thermoregulation, camouflage, social behavior, and economic value (16). In the high-altitude environments, such as the Tibetan Plateau, coat color also plays a critical role in adapting to extreme conditions like intense UV radiation and temperature fluctuations (17). Investigating the regulatory mechanisms underlying coat color can provide insights into hair follicle development, pigment synthesis, and deposition, which may benefit livestock breeding, high-quality fur production, and functional gene research (18). In this study, candidate genes and molecular markers related to the regulation of black and white coat color in Tibetan sheep were systematically identified through genome-wide selective sweep analysis, transcriptome sequencing, Sanger sequencing and protein structure prediction technology. Their potential regulatory mechanisms were further explored, providing a theoretical basis for advancing the study of livestock coat color genetics and supporting future molecular breeding strategies.

### 4.1 Identification of coat color–related genes and pathway

Classical pigmentation genes, including *MC1R*, *ASIP*, *TYRP1*, *KIT*, and *MITF*, have been widely reported to play key roles in sheep coat color regulation by directly influencing melanin deposition through tyrosine metabolism and melanogenesis (19–23). In this study, genome-wide selective sweep analysis and transcriptome profiling identified multiple candidate regions associated with black and white coat color in Tibetan sheep, including *KIT*, *MC1R*, and *MITF*. Both analytical approaches consistently revealed significant enrichment of the melanogenesis pathway, confirming the robustness of our strategy. To validate the transcriptome data, eight genes were selected for qRT-PCR based on their functional relevance in the melanogenesis pathway and their significant differential expression in RNA-seq analysis. These genes included key regulatory genes (*MITF*, *KIT*, *MC1R*) and downstream pigmentation-related genes, representing major components of the coat color regulatory network. The qRT-PCR expression patterns were highly consistent with RNA-seq results, confirming the reliability and reproducibility of the transcriptomic data. Although the number of validated genes was limited, their representative nature supports the robustness of our RNA-seq analysis.

In addition to pigmentation pathways, the transcriptomic analysis revealed significant enrichment in immune and inflammation-related pathways, including phagosome, Toll-like receptor (TLR) signaling, T cell receptor signaling, Th1/Th2/Th17 cell differentiation, and cytokine–cytokine receptor interaction. These findings are consistent with previous studies, highlighting the crucial role of the immune microenvironment in the hair follicle in regulating melanocyte survival, migration, and pigment synthesis (24). Immune cells, such as T cells and macrophages, are actively involved in modulating melanocyte activity and melanin production, especially under environmental stressors like ultraviolet (UV) exposure and temperature fluctuations (25). For example, the TLR signaling pathway, an essential component of the innate immune response, can activate local inflammatory reactions, promoting melanogenesis and helping to cope with UV-induced DNA damage by enhancing melanin synthesis (26). These immune responses are especially critical in high-altitude regions with strong UV radiation, as they not only aid in melanin synthesis but also enhance the skin’s UV-protective capacity, providing stronger protection (27). Thus, the immune system, through its regulation of melanocyte function in hair follicles, plays a vital role in responding to environmental stressors like UV radiation and temperature variation, thereby influencing coat color variation and adaptive evolution.

Additionally, arachidonic acid metabolism, branched chain amino acid biosynthesis, glutathione metabolism, mineral absorption and other pathways related to metabolism and nutrition were significantly enriched, which are closely related to substrate supply, redox homeostasis and keratin production required for pigment synthesis (28). For example, branched chain amino acids (BCAAs) (isoleucine, leucine, and valine) not only inhibit the melanogenesis, but also promote the repair of dermal collagen (29). Reduced glutathione plays a role by inhibiting tyrosinase activity, and its concentration reduction will stimulate epidermal melanocytes to produce melanin, resulting in skin pigmentation (30). Moreover, adequate supply of minerals such as copper and zinc is essential for the activity of key melanin producing enzymes such as tyrosinase (31). Selective sweep analysis also found that environmental adaptation and stress response pathways were significantly enriched, including thyroid hormone signaling, vasopressin regulated water reabsorption, aldosterone synthesis and secretion, calcium signaling and cGMP PKG signaling pathway. These pathways may be involved in physiological adaptation mechanisms to high altitude environments, such as osmoregulation and thermoregulation (32). The enrichment of metabolic and hormonal regulatory pathways such as insulin secretion, glucagon signal transduction and renin secretion suggests that there may be a physiological correlation between endocrine metabolic function and coat color (33). It is particularly noteworthy that the enrichment of DNA damage repair and genomic stability pathways, such as non-homologous end joining, mismatch repair and adhesion connection, indicated that coat color variation may be related to adaptation to high altitude ultraviolet radiation (34). Dark fur has stronger ultraviolet absorption capacity, potentially helping reduce the risks associated with UV-induced damage (35).

In conclusion, Tibetan sheep coat color is influenced not only by melanin synthesis but also by immune regulation, metabolic processes, DNA repair, and other mechanisms related to environmental adaptation. These findings provide a framework for understanding the molecular basis of coat color variation and may inform livestock breeding strategies in high-altitude regions.

### 4.2 Identification of molecular markers and mechanism analysis of coat color

We conducted transcriptome and selective sweep analyses and identified candidate genes related to Tibetan coat color, including *KIT, MC1R*, and *MITF*. Primers were designed for all exons of these genes, and 12 SNPs were screened using Sanger sequencing. Among them, *MC1R*-SNP1 (rs3508196008) and *MC1R*-SNP2 (rs409651063) were strongly associated with black coat color and exhibited a strong linkage relationship. These results were consistent with previous studies in Minxian Black Sheep, Norwegian Dala sheep, Chinese Tan sheep, Corriedale, Damara, Black Merino, Black Castellana, and Karakul sheep, supporting the use of these two SNPs as molecular markers for evaluating coat color (36–40). Although these mutations did not result in significant changes in protein secondary or 3D structures, nor involve alternative splicing, transcriptome analysis showed that *MC1R* expression in black GB sheep skin was significantly increased. Previous studies indicated that *MC1R* activation by α-melanocyte stimulating hormone (α-MSH) triggers the cAMP signaling pathway and activates key enzymes for melanin production, such as tyrosinase (41, 42). We speculate that these SNPs may enhance melanin synthesis by increasing *MC1R* transcription or improving α-MSH binding, thereby indirectly influencing coat color.

Recent comparative studies across other species, such as goats, cattle, and other sheep breeds, have provided important insights into the role of *MC1R* in coat color regulation. For instance, *MC1R* mutations have been shown to be significantly associated with coat color variation in goats, where specific alleles contribute to the expression of white or black coats (43). Similarly, in cattle, *MC1R* mutations have been linked to various coat colors, and its role in regulating melanin production is well-documented, especially in dairy and beef cattle breeds like Holstein and Angus (44, 45). Additionally, in other ovine breeds, such as the Corriedale and Brazilian Creole sheep, studies have also demonstrated how *MC1R* mutations influence wool color and skin pigmentation (46, 47). These findings further support the involvement of *MC1R* in coat color variation and highlight its potential for use as a molecular marker for livestock breeding.

## 5 Conclusion

This study identified genes and SNPs related to coat color in Tibetan sheep through selective sweep analysis, transcriptome analysis, and Sanger sequencing techniques. In addition, it was found that *MC1R*-SNP1 (rs3508196008) and *MC1R*-SNP2 (rs409651063) may regulate the coat color of Tibetan sheep by regulating the transcription level of this gene. They may be developed and utilized as molecular markers for coat color, providing new ideas for coat color genetics research and molecular breeding.

## Data Availability

The sequencing data from this study have been submitted to the NCBI SRA database. The transcriptome sequencing datasets were deposited under PRJNA1348487 (https://www.ncbi.nlm.nih.gov/bioproject/PRJNA1348487/), the whole-genome resequencing datasets of Guinan black fur sheep were deposited under PRJNA1353173 (https://www.ncbi.nlm.nih.gov/bioproject/PRJNA1353173/), and the whole genome sequencing data of Plateau Tibetan sheep sourced from our previous research PRJNA1111723 (https://www.ncbi.nlm.nih.gov/bioproject/PRJNA1111723/).

## Footnotes

^1^ https://www.ncbi.nlm.nih.gov/assembly/GCF_016772045.1

^2^ http://www.complextraitgenomics.com/software/gcta/

^3^ http://www.microbesonline.org/fasttree/

^4^ http://dalexander.github.io/admixture

^5^ http://geneontology.org

^6^ http://www.genome.ad.jp/kegg/

^7^ http://rnaseq-mats.sourceforge.net/index.html

^8^ http://espript.ibcp.fr/ESPript/ESPript/

^9^ https://swissmodel.expasy.org/
