# Supplementary material for: Revealing the genetic basis of coat color in Tibetan sheep through selective sweep and transcriptomic analyses
Source: Front Vet Sci. 2025 Nov 24;12:1711294. doi: 10.3389/fvets.2025.1711294 (PMC12682660; doi:10.3389/fvets.2025.1711294)
Supplement: Supplementary file 1 [file Table_1.docx]

***Supplementary Material***

**Revealing the genetic basis of coat color in Tibetan sheep through selective sweep and transcriptomic analyses**

**Xue Li^1^, Buying Han^1^, Dehong Tian^1^, Dehui Liu^1,2^, Wulong Ma^3^, Guangcai Bao^4^, Lei Wang^5^, Quanbang Pei^5^, Zian Zhang^5^, Kai Zhao^1*^**

^1^Key Laboratory of Adaptation and Evolution of Plateau Biota, Qinghai Provincial Key Laboratory of Animal Ecological Genomics, Northwest Institute of Plateau Biology, Chinese Academy of Sciences, Xining 810008, Qinghai, China

^2^University of Chinese Academy of Sciences, Beijing 100049, China

^3^Menyuan County Agriculture, Animal Husbandry and Water Conservancy Comprehensive Service Center, Menyuan 810300, Qinghai, China

^4^Qinghai Provincial Agricultural Project Guidance Service Center, Xining 810001, Qinghai, China

^5^Qinghai Sheep Breeding and Promotion Service Center, Gangcha 812300, Qinghai, China

***Corresponding author:**

Kai Zhao

E-mails: zhaokai@nwipb.cas.cn

**The supplementary file includes:**

Tables S1 to S18

Figure S1

**Table S1** The primer information of transcriptome validation

| **Primer name** | **Sequence (5’ to 3’)** |
| --- | --- |
| *FZD2*-F | GCTGCACCATTCTCTTCA |
| *FZD2*-R | GTGAGCGACAAGATGACC |
| *FZD3*-F | TCCATATCAGGTTACTCA |
| *FZD3*-R | TCTCGTTCACTATCTCTT |
| *GAPDH*-F | TGCCAAGTATGATGAGAT |
| *GAPDH*-R | TCAGTGTAGCCTAGAATG |
| *KIT*-F | GTCACAACAACCTTAGAAG |
| *KIT*-R | ATGCCTCATACTCAACAA |
| *LEF1*-F | GCTATCAACCAGATTCTA |
| *LEF1*-R | TCTTACCATAATTGTCTCT |
| *MC1R*-F | TTCTTCCTCCACCTCATC |
| *MC1R*-R | GTCAATCACAGAGTTACACAT |
| *PLCB2*-F | ACCAACTCCATCAATCCGGT |
| *PLCB2*-R | TGCGGTGTCCAAGAAACTTG |
| *TYR-*F | GACTTACTAGCCTGTTATG |
| *TYR*-R | CATTGTGGTAATCCTCTT |
| *TYRP1*-F | TGGACATAATAGACAATACA |
| *TYRP1*-R | CAATCTCAGGAATGCTAA |

**Table S2** The qPCR reaction system

| **Reagent** | **System** |
| --- | --- |
| 2×SYBR real-time PCR premixturereal-time PCR premixture | 10μL |
| 10uM Primer-F | 0.4 µL |
| 10uM Primer-R | 0.4 µL |
| cDNA | 1 μL |
| RNase free dH_2_O | Up to 20 μL |

**Table S3** The primer information of Sanger sequencing

| **Primer name** | **Sequence (5’ to 3’)** |
| --- | --- |
| *KIT*-01-F | GAGCCGGAACGTGGAACAGA |
| *KIT*-01-R | AGAAAAGGCTCTCCTACAAC |
| *KIT*-02-F | AAACAGAACTCTCTCACCAT |
| *KIT*-02-R | GGGCTAAACCATATTGTAAC |
| *KIT*-03-F | ACGACTGAAATGACTTAGCA |
| *KIT*-03-R | GCAGGTTTAATGACTATCCC |
| *KIT*-04-F | CTTGAAGGACACACAGTTTA |
| *KIT*-04-R | ACTACTGAATCACTTTGCCG |
| *KIT*-05-F | GTTCCAGTGACAGACTTGTC |
| *KIT*-05-R | AACACCTTCACAGTTACAGC |
| *KIT*-06-F | ACCAGCCTACTTGGCACTAC |
| *KIT*-06-R | ATAGGATGAGGTAGCAGTTA |
| *KIT*-07-F | ACAGCACAGGATGAGGGCGA |
| *KIT*-07-R | CTTCCAACCACCAAACCACG |
| *KIT*-08-F | CCTTTTTCGTGGTCAGACCT |
| *KIT*-08-R | AACTTGGCAATTAACTTCTG |
| *KIT*-09-F | GTGCTTTTCTTGCTCTCGGT |
| *KIT*-09-R | TAGCACAGAATGAAACTTAG |
| *KIT*-10-F | TCCTGCTGTGATGGGTGGTA |
| *KIT*-10-R | GACAGTAACTAGGTACGTCC |
| *KIT*-11-F | GATTGTACTCGTGCTTGGTA |
| *KIT*-11-R | GGACCCTGGCTCAACAGTAT |
| *KIT*-12-F | CCAGAGTCCCCTCTACAGTT |
| *KIT*-12-R | TTCATCAAGCACTGCGACTA |
| *KIT*-13-F | ACAGCAGTTGGAACATGAAG |
| *KIT*-13-R | ACACAGGCTCTGACTCCAAA |
| *KIT*-14-F | ACATCGTTCAAAGCGTAATC |
| *KIT*-14-R | AATTCTCCTGCTGTGACCTT |
| *KIT*-15-F | GGTCTTTGTGAGGTGCCCAT |
| *KIT*-15-R | AAACTCCCATGACCAACACT |
| *KIT*-16-F | TTGGACTATCACTTCTTATG |
| *KIT*-16-R | GTGACTACTGCTATGTACTC |
| *MC1R*-01-F | ATGCCTGTGCTCGGCTCCCA |
| *MC1R*-01-R | CCTCTTGGAGTGTCTTCCGG |
| *MC1R*-02-F | CTCCATCTTCTACGCCCTGC |
| *MC1R*-02-R | GCTATGAAGAAGCCAACCAG |
| *MITF*-01-F | CTGAGCTCTGCAAGGCTTGG |
| *MITF*-01-R | CTCGTTGGTGAACCCGTCCG |
| *MITF*-02-F | AGGTGGAATACTACTCTGCG |
| *MITF*-02-R | CATGTAGGAATCAATGGAGC |
| *MITF*-03-F | TTTCAAAGAGATGGACCGTT |
| *MITF*-04-F | CAGACTGATTTGATGGAAGT |
| *MITF*-04-R | ACACATCTCGGTTTTACAGA |
| *MITF*-05-F | CCTCATCACCCGTTAGCATT |
| *MITF*-05-R | ATACTAAGTTGGGCATCAGA |
| *MITF*-06-F | CAGTGTGCTGGGTAGAGTTC |
| *MITF*-06-R | TCAATGTTTCAACCACTGCG |
| *MITF*-07-F | TTGTACCTGTCTGTCGCTCC |
| *MITF*-07-R | TTGGGTAGTCAGGGATCAAG |
| *MITF*-08-F | ATTCAGGTTTTCATTGTCCC |
| *MITF*08-R | CCACCTCCAAAGCTGAACAG |
| *MITF*-09-F | AGATGTGAGTAGACTGTGTC |
| *MITF*-09-R | ATTTGCCACTCTGCACGTCT |
| *MITF*-10-F | TGACACGCCATTATCACTAC |
| *MITF*-10-R | ATCCTGGGCTATTGATAAAG |

**Table S4** The statistical results of sequencing data results

| **Sample** | **Reads Num** | **Total bases (bp)** | **N(%)** | **GC(%)** | **Q20(%)** | **Q30(%)** | **Sequencing depth** |
| --- | --- | --- | --- | --- | --- | --- | --- |
| GB01 | 182651750 | 27580414250 | 0.00 | 45.48 | 97.51 | 93.55 | 10.86 |
| GB02 | 205488816 | 31028811216 | 0.00 | 44.94 | 97.28 | 93.17 | 9.35 |
| GB03 | 244074022 | 36855177322 | 0.00 | 45.80 | 96.94 | 92.82 | 9.62 |
| GB04 | 249175144 | 37625446744 | 0.00 | 45.15 | 97.16 | 92.94 | 10.70 |
| GB05 | 155041278 | 23411232978 | 0.00 | 45.94 | 96.92 | 92.15 | 9.76 |
| GB06 | 188492708 | 28462398908 | 0.00 | 45.25 | 97.12 | 92.78 | 10.83 |
| GB07 | 197261426 | 29786475326 | 0.00 | 45.34 | 96.63 | 91.60 | 10.84 |
| GB08 | 194621644 | 29387868244 | 0.00 | 45.06 | 97.13 | 92.66 | 9.69 |
| GB09 | 160097312 | 24174694112 | 0.00 | 45.31 | 97.15 | 92.62 | 11.42 |
| GB10 | 194069498 | 29304494198 | 0.00 | 44.82 | 97.05 | 92.43 | 11.42 |
| GB11 | 189966206 | 28684897106 | 0.00 | 45.88 | 96.77 | 92.13 | 11.42 |
| GB12 | 177475236 | 26798760636 | 0.00 | 45.55 | 97.01 | 92.40 | 11.42 |
| GB13 | 172604138 | 26063224838 | 0.00 | 45.16 | 97.13 | 92.52 | 11.42 |
| GB14 | 188365022 | 28443118322 | 0.00 | 45.16 | 97.24 | 92.69 | 11.42 |
| GB15 | 195636030 | 29541040530 | 0.00 | 45.48 | 97.15 | 92.45 | 11.42 |
| GB16 | 173975894 | 26270359994 | 0.00 | 45.59 | 96.98 | 92.41 | 11.42 |
| GB17 | 190042516 | 28696419916 | 0.00 | 46.00 | 96.56 | 91.70 | 11.42 |
| GB18 | 173942434 | 26265307534 | 0.00 | 45.23 | 96.79 | 91.85 | 11.42 |
| GB19 | 165686306 | 25018632206 | 0.00 | 45.56 | 97.06 | 92.48 | 11.50 |
| GB20 | 202708168 | 30608933368 | 0.00 | 45.14 | 97.02 | 92.36 | 10.72 |
| PT01 | 201466634 | 30219995100 | 0.00 | 44.44 | 97.54 | 93.36 | 10.90 |
| PT02 | 187750038 | 28162505700 | 0.00 | 44.81 | 97.77 | 94.09 | 11.13 |
| PT03 | 224226482 | 33633972300 | 0.00 | 45.28 | 97.52 | 93.56 | 12.59 |
| PT04 | 194963632 | 29244544800 | 0.00 | 44.61 | 97.43 | 93.19 | 9.74 |
| PT05 | 195191168 | 29278675200 | 0.00 | 45.42 | 97.55 | 93.6 | 10.17 |
| PT06 | 198954750 | 29843212500 | 0.00 | 44.89 | 97.16 | 92.54 | 9.26 |
| PT07 | 195819412 | 29568731212 | 0.00 | 45.2 | 97.73 | 93.71 | 9.44 |
| PT08 | 183795352 | 27569302800 | 0.00 | 44.72 | 97.44 | 93.29 | 9.22 |
| PT09 | 183054986 | 27458247900 | 0.00 | 44.71 | 97.73 | 93.89 | 9.20 |
| PT10 | 223920344 | 33588051600 | 0.00 | 45.04 | 97.43 | 93.27 | 11.78 |
| PT11 | 188057082 | 28208562300 | 0.00 | 44.69 | 97.81 | 94.03 | 11.41 |
| PT12 | 172320726 | 26020429626 | 0.00 | 45.7 | 96.85 | 91.99 | 11.48 |
| PT13 | 214753014 | 32212952100 | 0.00 | 44.33 | 97.65 | 93.67 | 12.38 |
| PT14 | 177242838 | 26763668538 | 0.00 | 44.66 | 97.03 | 92.32 | 9.58 |
| PT15 | 189049366 | 28546454266 | 0.00 | 45.3 | 96.97 | 92.33 | 12.56 |
| PT16 | 186656896 | 28185191296 | 0.00 | 44.59 | 96.4 | 90.93 | 11.31 |
| PT17 | 180922132 | 27319241932 | 0.00 | 44.83 | 96.84 | 91.89 | 13.85 |
| PT18 | 202213550 | 30534246050 | 0.00 | 44.76 | 96.91 | 92.02 | 13.03 |
| PT19 | 168948950 | 25511291450 | 0.00 | 45.31 | 96.8 | 91.88 | 13.19 |
| PT20 | 191375862 | 28706379300 | 0.00 | 44.62 | 97.58 | 93.54 | 11.33 |
| Average | 191551469 | 28864584093 | 0.00 | 45.14 | 97.17 | 92.72 | 11.04 |

Sample: Sample name. Reads Num: Total number of reads. Total bases(bp): Total number of bases. N(%): Percentage of fuzzy bases. GC(%): GC content. Q20(%): Percentage of bases with base recognition accuracy above 99. Q30 (%): Percentage of bases with base recognition accuracy above 99.9

**Table S5** The statistical results of high-quality sequencing data

| **Sample** | **HQ_Reads** | **HQ_Reads (%)** | **HQ_Data (bp)** | **HQ_Data (**%**)** |
| --- | --- | --- | --- | --- |
| GB01 | 174299538 | 95.43 | 25996319143 | 94.26 |
| GB02 | 194402430 | 94.60 | 28986829613 | 93.42 |
| GB03 | 228352808 | 93.56 | 34029953474 | 92.33 |
| GB04 | 235819094 | 94.64 | 35126838580 | 93.36 |
| GB05 | 147097660 | 94.88 | 21874982109 | 93.44 |
| GB06 | 178317134 | 94.60 | 26567746400 | 93.34 |
| GB07 | 185684422 | 94.13 | 27593979916 | 92.64 |
| GB08 | 184524856 | 94.81 | 27485285096 | 93.53 |
| GB09 | 152434160 | 95.21 | 22689309396 | 93.86 |
| GB10 | 183827222 | 94.72 | 27370664987 | 93.40 |
| GB11 | 178241868 | 93.83 | 26517208329 | 92.44 |
| GB12 | 168252676 | 94.80 | 25042073307 | 93.44 |
| GB13 | 164447538 | 95.27 | 24482333192 | 93.93 |
| GB14 | 179862134 | 95.49 | 26793425472 | 94.20 |
| GB15 | 186720048 | 95.44 | 27791897514 | 94.08 |
| GB16 | 164130934 | 94.34 | 24440315587 | 93.03 |
| GB17 | 176997544 | 93.14 | 26321897087 | 91.73 |
| GB18 | 164271760 | 94.44 | 24431949996 | 93.02 |
| GB19 | 157311126 | 94.95 | 23401944658 | 93.54 |
| GB20 | 192319366 | 94.87 | 28626195976 | 93.52 |
| PT01 | 194060136 | 96.32 | 28730307128 | 95.07 |
| PT02 | 181665066 | 96.76 | 26905834839 | 95.54 |
| PT03 | 215483522 | 96.10 | 31874605747 | 94.77 |
| PT04 | 187318822 | 96.08 | 27712308528 | 94.76 |
| PT05 | 187677230 | 96.15 | 27767211515 | 94.84 |
| PT06 | 190332358 | 95.67 | 28123647822 | 94.24 |
| PT07 | 190298644 | 97.18 | 28383670741 | 95.99 |
| PT08 | 176548912 | 96.06 | 26107704370 | 94.70 |
| PT09 | 177012066 | 96.70 | 26228307475 | 95.52 |
| PT10 | 214928620 | 95.98 | 31782567457 | 94.62 |
| PT11 | 182283260 | 96.93 | 27021857533 | 95.79 |
| PT12 | 164468874 | 95.44 | 24383476618 | 93.71 |
| PT13 | 207463798 | 96.61 | 30736829771 | 95.42 |
| PT14 | 169908938 | 95.86 | 25206115467 | 94.18 |
| PT15 | 180720660 | 95.59 | 26804602981 | 93.90 |
| PT16 | 176673834 | 94.65 | 26142501677 | 92.75 |
| PT17 | 172768558 | 95.49 | 25618760459 | 93.78 |
| PT18 | 193519646 | 95.70 | 28692089468 | 93.97 |
| PT19 | 161143272 | 95.38 | 23882880160 | 93.62 |
| PT20 | 184694198 | 96.51 | 27356912853 | 95.30 |
| Average | 182657118 | 95.36 | 27125833561 | 93.97 |

Sample: Sample name. HQ reads: Number of high-quality reads. HQ reads (%): Percentage of high-quality reads to original reads. HQ Bases (bp): Number of bases for high-quality reads. HQ Bases(%): Percentage of base number of high-quality reads to total number of original bases

**Table S6** The results of the read mapping

| **Sample** | **Total_reads** | **Mapped_reads** | **Mapping_rate (%)** |
| --- | --- | --- | --- |
| GB01 | 174663211 | 174481306 | 99.90 |
| GB02 | 194814833 | 194581901 | 99.88 |
| GB03 | 228824434 | 228335718 | 99.79 |
| GB04 | 236328353 | 235961904 | 99.84 |
| GB05 | 147405947 | 147255986 | 99.90 |
| GB06 | 178682096 | 178515013 | 99.91 |
| GB07 | 186086614 | 185855888 | 99.88 |
| GB08 | 184918866 | 184668510 | 99.86 |
| GB09 | 152769241 | 152602039 | 99.89 |
| GB10 | 184215133 | 183938990 | 99.85 |
| GB11 | 178652402 | 178153892 | 99.72 |
| GB12 | 168641781 | 168327227 | 99.81 |
| GB13 | 164839363 | 164504140 | 99.80 |
| GB14 | 180293395 | 179958682 | 99.81 |
| GB15 | 187159914 | 186649625 | 99.73 |
| GB16 | 164514976 | 164214036 | 99.82 |
| GB17 | 177402599 | 176906259 | 99.72 |
| GB18 | 164652753 | 164350095 | 99.82 |
| GB19 | 157694151 | 157185831 | 99.68 |
| GB20 | 192774973 | 192371616 | 99.79 |
| PT01 | 194510453 | 194192678 | 99.84 |
| PT02 | 182103796 | 181707672 | 99.78 |
| PT03 | 215993790 | 215495314 | 99.77 |
| PT04 | 187754437 | 187492438 | 99.86 |
| PT05 | 188124783 | 187625595 | 99.73 |
| PT06 | 190761346 | 190431856 | 99.83 |
| PT07 | 190717222 | 190250430 | 99.76 |
| PT08 | 176979491 | 176349501 | 99.64 |
| PT09 | 177431537 | 177134636 | 99.83 |
| PT10 | 215446802 | 214790243 | 99.70 |
| PT11 | 182665587 | 182419378 | 99.87 |
| PT12 | 164801665 | 164475155 | 99.80 |
| PT13 | 207901991 | 207654321 | 99.88 |
| PT14 | 170260125 | 170144855 | 99.93 |
| PT15 | 181081567 | 180899113 | 99.90 |
| PT16 | 177046550 | 176945509 | 99.94 |
| PT17 | 173119549 | 172886966 | 99.87 |
| PT18 | 193910674 | 193453761 | 99.76 |
| PT19 | 161477071 | 161288783 | 99.88 |
| PT20 | 185079939 | 184900479 | 99.90 |
| Average | 183062585 | 182733934 | 99.82 |

Sample: Sample name. Total reads: Total reads quantity. Mapped reads: Reads number aligned to the reference genome (including single-sided and double-ended alignments). Mapping rate: Alignment rate, which is the percentage of reads on the reference genome that are aligned to the total number of reads

**Table S7** The results of SNPs annotation.

| **Type** | **Number** | **Percentage (%)** |
| --- | --- | --- |
| exonic total | 80717 | 0.87 |
| synonymous SNV | 51974 | 0.56 |
| nonsynonymous SNV | 26188 | 0.28 |
| stopgain | 266 | 0.01 |
| stoploss | 57 | 0.0 |
| unknown | 2232 | 0.02 |
| splicing | 247 | 0.0 |
| ncRNA total | 2078 | 0.02 |
| ncRNA_exonic | 844 | 0.01 |
| ncRNA_splicing | 2 | 0.0 |
| ncRNA_exonic;splicing | 0 | 0.0 |
| ncRNA_intronic | 1232 | 0.01 |
| intronic | 3473133 | 37.43 |
| intergenic | 5497549 | 59.25 |
| UTR5 | 32793 | 0.35 |
| UTR3 | 72137 | 0.78 |
| UTR5;UTR3 | 138 | 0.0 |
| upstream | 58605 | 0.63 |
| downstream | 59919 | 0.65 |
| upstream;downstream | 2024 | 0.02 |
| Total | 9279340 | 100.0 |

Type: The type of SNPs. Number: The number of SNPs of this type. Percentage (%): The proportion of this type SNPs of to the total number of SNPs

**Table S8** The results of InDels annotation.

| **Type** | **Number** | **Percentage (%)** |
| --- | --- | --- |
| exonic total | 6267 | 0.15 |
| frameshift deletion | 1811 | 0.04 |
| frameshift insertion | 1271 | 0.03 |
| nonframeshift deletion | 1569 | 0.04 |
| nonframeshift insertion | 957 | 0.02 |
| nonframeshift substitution | 0 | 0.00 |
| stopgain | 74 | 0.00 |
| stoploss | 8 | 0.00 |
| unknown | 577 | 0.01 |
| splicing | 327 | 0.01 |
| ncRNA total | 1149 | 0.03 |
| ncRNA_exonic | 276 | 0.01 |
| ncRNA_exonic;splicing | 0 | 0.00 |
| ncRNA_splicing | 1 | 0.00 |
| ncRNA_intronic | 872 | 0.02 |
| ncRNA_UTR5 | 0 | 0.00 |
| intronic | 1506226 | 36.88 |
| intergenic | 2460475 | 60.24 |
| UTR5 | 13407 | 0.33 |
| UTR3 | 36081 | 0.88 |
| UTR5;UTR3 | 61 | 0.00 |
| upstream | 29456 | 0.72 |
| downstream | 29829 | 0.73 |
| upstream;downstream | 1126 | 0.03 |
| Total | 4084404 | 100.00 |

Type: The type of InDels. Number: The number of InDels of this type. Percentage (%): The proportion of this type of InDels to the total number

**Table S9** Selective sweep analysis of Plateau Tibetan sheep and Guinan black fur sheep (partial results).

| **CHROM** | | **Pi** | **Fst** | **color** | **gene** |
| --- | --- | --- | --- | --- | --- |
| NC_056068.1_2945001 | | -3.40 | 0.11 | Selectedregion(GB region) | gene-LOC101117272 |
| NC_056068.1_2940001 | | -3.19 | 0.09 | Selectedregion(GB region) | gene-LOC101117272 |
| NC_056068.1_2970001 | | -2.70 | 0.11 | Selectedregion(GB region) | - |
| NC_056068.1_2935001 | | -2.68 | 0.08 | Selectedregion(GB region) | gene-LOC101117272 |
| NC_056068.1_2965001 | | -2.64 | 0.11 | Selectedregion(GB region) | - |
| NC_056058.1_83185001 | | -2.50 | 0.14 | Selectedregion(GB region) | - |
| NC_056064.1_27740001 | | -2.45 | 0.08 | Selectedregion(GB region) | gene-ARHGEF15 |
| NC_056066.1_15735001 | | -2.39 | 0.16 | Selectedregion(GB region) | - |
| NC_056064.1_27745001 | | -2.39 | 0.09 | Selectedregion(GB region) | gene-ARHGEF15 |
| NC_056080.1_85460001 | | -2.38 | 0.21 | Selectedregion(GB region) | - |
| NC_056068.1_2930001 | | -2.36 | 0.08 | Selectedregion(GB region) | gene-LOC101117272 |
| NC_056068.1_2930001 | | -2.36 | 0.08 | Selectedregion(GB region) | gene-LOC101117013 |
| NC_056068.1_2980001 | | -2.34 | 0.09 | Selectedregion(GB region) | - |
| NC_056066.1_15730001 | | -2.32 | 0.16 | Selectedregion(GB region) | gene-TRNAW-CCA-155 |
| NC_056068.1_2925001 | | -2.32 | 0.08 | Selectedregion(GB region) | gene-LOC101117272 |
| NC_056068.1_2925001 | | -2.32 | 0.08 | Selectedregion(GB region) | gene-LOC101117013 |
| NC_056068.1_2985001 | | -2.15 | 0.08 | Selectedregion(GB region) | - |
| NC_056064.1_27750001 | | -2.14 | 0.08 | Selectedregion(GB region) | gene-ARHGEF15 |
| NC_056064.1_27750001 | | -2.14 | 0.08 | Selectedregion(GB region) | gene-ODF4 |
| NC_056068.1_2905001 | | -2.14 | 0.09 | Selectedregion(GB region) | gene-LOC101117013 |
| NC_056080.1_50665001 | | -2.12 | 0.10 | Selectedregion(GB region) | - |
| NC_056068.1_2975001 | | -2.12 | 0.09 | Selectedregion(GB region) | - |
| NC_056068.1_2910001 | | -2.10 | 0.08 | Selectedregion(GB region) | gene-LOC101117272 |
| NC_056068.1_2910001 | | -2.10 | 0.08 | Selectedregion(GB region) | gene-LOC101117013 |
| NC_056059.1_72110001 | | -2.09 | 0.12 | Selectedregion(GB region) | gene-CEP135 |
| NC_056074.1_28480001 | | -2.09 | 0.14 | Selectedregion(GB region) | - |
| NC_056080.1_50675001 | | -2.08 | 0.09 | Selectedregion(GB region) | gene-LOC114111329 |
| NC_056080.1_50670001 | | -2.06 | 0.10 | Selectedregion(GB region) | gene-LOC114111329 |
| NC_056059.1_96540001 | | -2.02 | 0.14 | Selectedregion(GB region) | gene-BMP3 |
| NC_056080.1_50625001 | | -2.01 | 0.14 | Selectedregion(GB region) | gene-LOC114111342 |
| NC_056080.1_50625001 | | -2.01 | 0.14 | Selectedregion(GB region) | gene-LOC121818363 |
| NC_056080.1_85455001 | | -2.01 | 0.21 | Selectedregion(GB region) | - |
| NC_056059.1_96535001 | | -1.98 | 0.15 | Selectedregion(GB region) | gene-BMP3 |
| NC_056080.1_26110001 | | -1.98 | 0.14 | Selectedregion(GB region) | - |
| NC_056064.1_550001 | | -1.96 | 0.24 | Selectedregion(GB region) | gene-CA10 |
| NC_056080.1_26105001 | | -1.95 | 0.14 | Selectedregion(GB region) | - |
| NC_056060.1_50355001 | | -1.91 | 0.13 | Selectedregion(GB region) | gene-ALDH1A2 |
| NC_056064.1_555001 | | -1.90 | 0.23 | Selectedregion(GB region) | gene-CA10 |
| NC_056057.1_59775001 | | -1.90 | 0.14 | Selectedregion(GB region) | - |
| NC_056064.1_27735001 | | -1.86 | 0.07 | Selectedregion(GB region) | gene-ARHGEF15 |
| NC_056066.1_15725001 | | -1.86 | 0.14 | Selectedregion(GB region) | gene-TRNAW-CCA-155 |
| NC_056066.1_15725001 | | -1.86 | 0.14 | Selectedregion(GB region) | gene-USP6NL |
| NC_056057.1_1030001 | | -1.83 | 0.12 | Selectedregion(GB region) | - |
| NC_056057.1_1040001 | | -1.83 | 0.12 | Selectedregion(GB region) | - |
| NC_056055.1_31495001 | | -1.81 | 0.12 | Selectedregion(GB region) | gene-AOPEP |
| NC_056080.1_50765001 | | -1.80 | 0.10 | Selectedregion(GB region) | gene-LOC114111461 |
| NC_056080.1_50860001 | | -1.80 | 0.09 | Selectedregion(GB region) | - |
| NC_056064.1_27755001 | | -1.79 | 0.08 | Selectedregion(GB region) | gene-ODF4 |
| NC_056066.1_58740001 | | -1.78 | 0.12 | Selectedregion(GB region) | - |
| NC_056068.1_2900001 | | -1.78 | 0.10 | Selectedregion(GB region) | gene-LOC101117013 |
| NC_056055.1_186570001 | 3.38 | 0.13 | Selectedregion(PT region) | gene-TFCP2L1 |  |
| NC_056055.1_186565001 | 3.33 | 0.13 | Selectedregion(PT region) | gene-TFCP2L1 |  |
| NC_056055.1_186575001 | 3.30 | 0.13 | Selectedregion(PT region) | gene-TFCP2L1 |  |
| NC_056057.1_49035001 | 2.76 | 0.11 | Selectedregion(PT region) | - |  |
| NC_056055.1_186560001 | 2.61 | 0.12 | Selectedregion(PT region) | gene-TFCP2L1 |  |
| NC_056055.1_186580001 | 2.53 | 0.14 | Selectedregion(PT region) | gene-TFCP2L1 |  |
| NC_056057.1_49040001 | 2.49 | 0.12 | Selectedregion(PT region) | - |  |
| NC_056068.1_73400001 | 2.44 | 0.16 | Selectedregion(PT region) | - |  |
| NC_056068.1_73395001 | 2.40 | 0.15 | Selectedregion(PT region) | - |  |
| NC_056057.1_49045001 | 2.37 | 0.12 | Selectedregion(PT region) | - |  |
| NC_056057.1_49010001 | 2.34 | 0.11 | Selectedregion(PT region) | - |  |
| NC_056080.1_124335001 | 2.24 | 0.08 | Selectedregion(PT region) | - |  |
| NC_056072.1_54340001 | 2.14 | 0.15 | Selectedregion(PT region) | gene-ATP2B2 |  |
| NC_056077.1_11100001 | 2.05 | 0.11 | Selectedregion(PT region) | gene-GSPT1 |  |
| NC_056077.1_11100001 | 2.05 | 0.11 | Selectedregion(PT region) | gene-LOC121817877 |  |
| NC_056072.1_54335001 | 2.04 | 0.14 | Selectedregion(PT region) | gene-ATP2B2 |  |
| NC_056057.1_49110001 | 2.02 | 0.07 | Selectedregion(PT region) | - |  |
| NC_056055.1_186585001 | 1.96 | 0.13 | Selectedregion(PT region) | gene-TFCP2L1 |  |
| NC_056080.1_124340001 | 1.95 | 0.09 | Selectedregion(PT region) | - |  |
| NC_056068.1_73405001 | 1.95 | 0.13 | Selectedregion(PT region) | - |  |
| NC_056063.1_7455001 | 1.87 | 0.09 | Selectedregion(PT region) | - |  |
| NC_056054.1_121985001 | 1.87 | 0.13 | Selectedregion(PT region) | - |  |
| NC_056063.1_7450001 | 1.86 | 0.09 | Selectedregion(PT region) | - |  |
| NC_056080.1_133285001 | 1.85 | 0.12 | Selectedregion(PT region) | gene-ARMCX2 |  |
| NC_056057.1_49105001 | 1.83 | 0.06 | Selectedregion(PT region) | - |  |
| NC_056067.1_38575001 | 1.82 | 0.09 | Selectedregion(PT region) | gene-DHODH |  |
| NC_056072.1_54345001 | 1.82 | 0.14 | Selectedregion(PT region) | gene-ATP2B2 |  |
| NC_056067.1_38570001 | 1.79 | 0.09 | Selectedregion(PT region) | - |  |
| NC_056071.1_31075001 | 1.78 | 0.13 | Selectedregion(PT region) | gene-SIN3A |  |
| NC_056054.1_121990001 | 1.78 | 0.12 | Selectedregion(PT region) | - |  |
| NC_056057.1_49100001 | 1.77 | 0.06 | Selectedregion(PT region) | - |  |
| NC_056077.1_11115001 | 1.77 | 0.12 | Selectedregion(PT region) | gene-LOC121817877 |  |
| NC_056056.1_90185001 | 1.75 | 0.05 | Selectedregion(PT region) | - |  |
| NC_056066.1_49580001 | 1.71 | 0.09 | Selectedregion(PT region) | - |  |
| NC_056057.1_49005001 | 1.68 | 0.09 | Selectedregion(PT region) | - |  |
| NC_056057.1_49075001 | 1.67 | 0.07 | Selectedregion(PT region) | - |  |
| NC_056068.1_73390001 | 1.66 | 0.12 | Selectedregion(PT region) | - |  |
| NC_056063.1_44345001 | 1.66 | 0.09 | Selectedregion(PT region) | - |  |
| NC_056066.1_49585001 | 1.66 | 0.09 | Selectedregion(PT region) | - |  |
| NC_056077.1_11105001 | 1.66 | 0.10 | Selectedregion(PT region) | gene-GSPT1 |  |
| NC_056077.1_11105001 | 1.66 | 0.10 | Selectedregion(PT region) | gene-LOC121817877 |  |
| NC_056063.1_32055001 | 1.66 | 0.08 | Selectedregion(PT region) | gene-FLT1 |  |
| NC_056055.1_75125001 | 1.66 | 0.07 | Selectedregion(PT region) | - |  |
| NC_056066.1_49575001 | 1.66 | 0.08 | Selectedregion(PT region) | - |  |
| NC_056080.1_124365001 | 1.66 | 0.06 | Selectedregion(PT region) | - |  |
| NC_056063.1_32060001 | 1.65 | 0.08 | Selectedregion(PT region) | gene-FLT1 |  |
| NC_056063.1_32035001 | 1.65 | 0.09 | Selectedregion(PT region) | gene-FLT1 |  |
| NC_056055.1_186555001 | 1.65 | 0.11 | Selectedregion(PT region) | gene-TFCP2L1 |  |
| NC_056072.1_21360001 | 1.65 | 0.10 | Selectedregion(PT region) | gene-LOC105603426 |  |
| NC_056055.1_75115001 | 1.64 | 0.08 | Selectedregion(PT region) | gene-KDM4C |  |

**Table S10** The KEGG enrichment analysis of genes in selective sweep regions of Plateau Tibetan sheep and Guinan black fur sheep (*P* < 0.05).

| **PathwayID** | **Pathway** | **list_number** | **total_number** | ***P*-value** |
| --- | --- | --- | --- | --- |
| ko00790 | Folate biosynthesis | 12 | 42 | 0.0004 |
| ko04928 | Parathyroid hormone synthesis, secretion and action | 22 | 114 | 0.0011 |
| ko04927 | Cortisol synthesis and secretion | 15 | 67 | 0.0014 |
| ko04913 | Ovarian steroidogenesis | 14 | 63 | 0.0022 |
| ko03450 | Non-homologous end-joining | 5 | 13 | 0.0054 |
| ko04310 | Wnt signaling pathway | 27 | 169 | 0.0056 |
| ko03020 | RNA polymerase | 8 | 30 | 0.0061 |
| ko04911 | Insulin secretion | 16 | 87 | 0.0081 |
| ko04020 | Calcium signaling pathway | 36 | 249 | 0.0082 |
| ko04270 | Vascular smooth muscle contraction | 22 | 136 | 0.0102 |
| ko04922 | Glucagon signaling pathway | 18 | 105 | 0.0107 |
| ko04919 | Thyroid hormone signaling pathway | 20 | 123 | 0.0131 |
| ko04022 | cGMP-PKG signaling pathway | 25 | 165 | 0.0145 |
| ko04916 | Melanogenesis | 17 | 101 | 0.0154 |
| ko05200 | Pathways in cancer | 67 | 541 | 0.0169 |
| ko03430 | Mismatch repair | 6 | 23 | 0.0188 |
| ko04138 | Autophagy - yeast | 14 | 80 | 0.0193 |
| ko04925 | Aldosterone synthesis and secretion | 16 | 96 | 0.0201 |
| ko04730 | Long-term depression | 11 | 59 | 0.0233 |
| ko05412 | Arrhythmogenic right ventricular cardiomyopathy | 14 | 82 | 0.0235 |
| ko04720 | Long-term potentiation | 12 | 67 | 0.0246 |
| ko00965 | Betalain biosynthesis | 2 | 3 | 0.0259 |
| ko04611 | Platelet activation | 19 | 124 | 0.0273 |
| ko04725 | Cholinergic synapse | 17 | 109 | 0.0306 |
| ko04520 | Adherens junction | 15 | 93 | 0.0312 |
| ko04261 | Adrenergic signaling in cardiomyocytes | 22 | 151 | 0.0312 |
| ko04962 | Vasopressin-regulated water reabsorption | 9 | 47 | 0.0327 |
| ko05132 | Salmonella infection | 34 | 257 | 0.0337 |
| ko04728 | Dopaminergic synapse | 19 | 127 | 0.0340 |
| ko04924 | Renin secretion | 12 | 71 | 0.0369 |
| ko04360 | Axon guidance | 25 | 180 | 0.0381 |
| ko04935 | Growth hormone synthesis, secretion and action | 18 | 121 | 0.0403 |

**Table S11** The GO enrichment analysis of genes in selective sweep regions of Plateau Tibetan sheep and Guinan black fur sheep (*P* < 0.01).

| **Category** | **GO.ID** | **Term** | **list** | **Total** | **Pvalue** |
| --- | --- | --- | --- | --- | --- |
| BP | GO:0060255 | regulation of macromolecule metabolic process | 376 | 3023 | 0.0001 |
| MF | GO:0032138 | single base insertion or deletion binding | 4 | 4 | 0.0001 |
| BP | GO:0051173 | positive regulation of nitrogen compound metabolic process | 193 | 1427 | 0.0001 |
| MF | GO:0030695 | GTPase regulator activity | 55 | 318 | 0.0001 |
| MF | GO:0060589 | nucleoside-triphosphatase regulator activity | 55 | 318 | 0.0001 |
| MF | GO:0070273 | phosphatidylinositol-4-phosphate binding | 8 | 17 | 0.0001 |
| CC | GO:0005790 | smooth endoplasmic reticulum | 6 | 10 | 0.0002 |
| CC | GO:0032300 | mismatch repair complex | 5 | 7 | 0.0002 |
| CC | GO:0099081 | supramolecular polymer | 72 | 466 | 0.0002 |
| MF | GO:0005085 | guanyl-nucleotide exchange factor activity | 34 | 173 | 0.0002 |
| CC | GO:0099512 | supramolecular fiber | 71 | 461 | 0.0002 |
| BP | GO:0050714 | positive regulation of protein secretion | 16 | 58 | 0.0003 |
| MF | GO:0030554 | adenyl nucleotide binding | 166 | 1236 | 0.0003 |
| CC | GO:0030286 | dynein complex | 12 | 39 | 0.0003 |
| MF | GO:0032559 | adenyl ribonucleotide binding | 165 | 1232 | 0.0004 |
| BP | GO:0045913 | positive regulation of carbohydrate metabolic process | 11 | 34 | 0.0005 |
| MF | GO:0032135 | DNA insertion or deletion binding | 4 | 5 | 0.0006 |
| BP | GO:0010919 | regulation of inositol phosphate biosynthetic process | 5 | 8 | 0.0006 |
| BP | GO:0062013 | positive regulation of small molecule metabolic process | 16 | 62 | 0.0006 |
| BP | GO:0051096 | positive regulation of helicase activity | 4 | 5 | 0.0006 |
| MF | GO:0003677 | DNA binding | 172 | 1302 | 0.0006 |
| MF | GO:0005524 | ATP binding | 160 | 1200 | 0.0006 |
| BP | GO:0009893 | positive regulation of metabolic process | 240 | 1877 | 0.0006 |
| MF | GO:0047834 | D-threo-aldose 1-dehydrogenase activity | 7 | 16 | 0.0007 |
| BP | GO:0050796 | regulation of insulin secretion | 17 | 69 | 0.0007 |
| BP | GO:0048468 | cell development | 160 | 1191 | 0.0007 |
| BP | GO:0032958 | inositol phosphate biosynthetic process | 7 | 16 | 0.0007 |
| CC | GO:0098793 | presynapse | 36 | 204 | 0.0007 |
| BP | GO:0048519 | negative regulation of biological process | 352 | 2880 | 0.0008 |
| BP | GO:0090277 | positive regulation of peptide hormone secretion | 12 | 41 | 0.0008 |
| BP | GO:0010604 | positive regulation of macromolecule metabolic process | 219 | 1703 | 0.0008 |
| CC | GO:0005874 | microtubule | 36 | 206 | 0.0009 |
| CC | GO:0016605 | PML body | 14 | 55 | 0.0010 |
| BP | GO:0043666 | regulation of phosphoprotein phosphatase activity | 10 | 31 | 0.0010 |
| BP | GO:0046887 | positive regulation of hormone secretion | 14 | 53 | 0.0010 |
| BP | GO:0048523 | negative regulation of cellular process | 314 | 2548 | 0.0010 |
| CC | GO:0000922 | spindle pole | 19 | 87 | 0.0010 |
| BP | GO:0002793 | positive regulation of peptide secretion | 12 | 42 | 0.0011 |
| CC | GO:0030015 | CCR4-NOT core complex | 3 | 3 | 0.0011 |
| MF | GO:0032404 | mismatch repair complex binding | 5 | 9 | 0.0011 |
| CC | GO:0034704 | calcium channel complex | 12 | 44 | 0.0011 |
| BP | GO:0030900 | forebrain development | 40 | 230 | 0.0012 |
| MF | GO:0032142 | single guanine insertion binding | 3 | 3 | 0.0012 |
| BP | GO:0032957 | inositol trisphosphate metabolic process | 5 | 9 | 0.0012 |
| BP | GO:0051095 | regulation of helicase activity | 5 | 9 | 0.0012 |
| BP | GO:0046068 | cGMP metabolic process | 3 | 3 | 0.0012 |
| BP | GO:0010468 | regulation of gene expression | 291 | 2350 | 0.0012 |
| MF | GO:0005262 | calcium channel activity | 16 | 67 | 0.0013 |
| BP | GO:0009187 | cyclic nucleotide metabolic process | 8 | 22 | 0.0013 |
| BP | GO:0016601 | Rac protein signal transduction | 8 | 22 | 0.0013 |
| MF | GO:0008092 | cytoskeletal protein binding | 87 | 605 | 0.0013 |
| BP | GO:0051171 | regulation of nitrogen compound metabolic process | 325 | 2658 | 0.0014 |
| BP | GO:0030073 | insulin secretion | 20 | 92 | 0.0014 |
| CC | GO:0060076 | excitatory synapse | 8 | 23 | 0.0014 |
| CC | GO:0042734 | presynaptic membrane | 12 | 45 | 0.0014 |
| BP | GO:0080090 | regulation of primary metabolic process | 336 | 2761 | 0.0015 |
| BP | GO:0009214 | cyclic nucleotide catabolic process | 4 | 6 | 0.0016 |
| BP | GO:0032959 | inositol trisphosphate biosynthetic process | 4 | 6 | 0.0016 |
| BP | GO:0060732 | positive regulation of inositol phosphate biosynthetic process | 4 | 6 | 0.0016 |
| BP | GO:0031325 | positive regulation of cellular metabolic process | 182 | 1402 | 0.0016 |
| CC | GO:0098590 | plasma membrane region | 85 | 610 | 0.0016 |
| BP | GO:0043085 | positive regulation of catalytic activity | 85 | 586 | 0.0016 |
| CC | GO:0005654 | nucleoplasm | 290 | 2447 | 0.0017 |
| BP | GO:0007417 | central nervous system development | 75 | 506 | 0.0017 |
| MF | GO:0016887 | ATP hydrolysis activity | 19 | 88 | 0.0017 |
| BP | GO:2000112 | regulation of cellular macromolecule biosynthetic process | 41 | 242 | 0.0017 |
| BP | GO:0097305 | response to alcohol | 15 | 62 | 0.0017 |
| MF | GO:1901363 | heterocyclic compound binding | 443 | 3782 | 0.0018 |
| CC | GO:0098978 | glutamatergic synapse | 33 | 192 | 0.0019 |
| BP | GO:0045935 | positive regulation of nucleobase-containing compound metabolic process | 120 | 878 | 0.0019 |
| MF | GO:0097159 | organic cyclic compound binding | 446 | 3813 | 0.0020 |
| CC | GO:0042641 | actomyosin | 10 | 35 | 0.0020 |
| BP | GO:1903580 | positive regulation of ATP metabolic process | 6 | 14 | 0.0020 |
| BP | GO:1902932 | positive regulation of alcohol biosynthetic process | 5 | 10 | 0.0021 |
| BP | GO:0050790 | regulation of catalytic activity | 122 | 899 | 0.0022 |
| MF | GO:0051020 | GTPase binding | 34 | 196 | 0.0022 |
| BP | GO:0030182 | neuron differentiation | 101 | 724 | 0.0022 |
| CC | GO:0001725 | stress fiber | 9 | 30 | 0.0023 |
| CC | GO:0097517 | contractile actin filament bundle | 9 | 30 | 0.0023 |
| MF | GO:0015175 | neutral amino acid transmembrane transporter activity | 8 | 24 | 0.0023 |
| BP | GO:0031328 | positive regulation of cellular biosynthetic process | 116 | 850 | 0.0023 |
| BP | GO:0009891 | positive regulation of biosynthetic process | 118 | 867 | 0.0024 |
| MF | GO:0015085 | calcium ion transmembrane transporter activity | 18 | 84 | 0.0024 |
| MF | GO:0003676 | nucleic acid binding | 260 | 2123 | 0.0024 |
| BP | GO:0010605 | negative regulation of macromolecule metabolic process | 167 | 1285 | 0.0024 |
| CC | GO:0030424 | axon | 38 | 234 | 0.0025 |
| MF | GO:0043565 | sequence-specific DNA binding | 105 | 769 | 0.0026 |
| CC | GO:0099061 | integral component of postsynaptic density membrane | 7 | 20 | 0.0026 |
| BP | GO:0032501 | multicellular organismal process | 466 | 3972 | 0.0027 |
| BP | GO:0072507 | divalent inorganic cation homeostasis | 31 | 175 | 0.0029 |
| BP | GO:0010921 | regulation of phosphatase activity | 17 | 78 | 0.0029 |
| MF | GO:0120013 | lipid transfer activity | 8 | 25 | 0.0031 |
| BP | GO:0035774 | positive regulation of insulin secretion involved in cellular response to glucose stimulus | 6 | 15 | 0.0031 |
| BP | GO:1901021 | positive regulation of calcium ion transmembrane transporter activity | 6 | 15 | 0.0031 |
| MF | GO:0045296 | cadherin binding | 7 | 20 | 0.0031 |
| MF | GO:0031267 | small GTPase binding | 31 | 178 | 0.0032 |
| BP | GO:0010557 | positive regulation of macromolecule biosynthetic process | 109 | 800 | 0.0033 |
| BP | GO:0023052 | signaling | 385 | 3239 | 0.0034 |
| BP | GO:0006357 | regulation of transcription by RNA polymerase II | 111 | 817 | 0.0034 |
| BP | GO:0032469 | endoplasmic reticulum calcium ion homeostasis | 7 | 20 | 0.0034 |
| BP | GO:0045471 | response to ethanol | 7 | 20 | 0.0034 |
| BP | GO:0072503 | cellular divalent inorganic cation homeostasis | 29 | 162 | 0.0034 |
| MF | GO:0030983 | mismatched DNA binding | 5 | 11 | 0.0034 |
| BP | GO:0003407 | neural retina development | 11 | 42 | 0.0036 |
| BP | GO:0071692 | protein localization to extracellular region | 30 | 170 | 0.0036 |
| BP | GO:0000122 | negative regulation of transcription by RNA polymerase II | 53 | 345 | 0.0037 |
| CC | GO:0032432 | actin filament bundle | 9 | 32 | 0.0037 |
| CC | GO:0030054 | cell junction | 134 | 1056 | 0.0037 |
| CC | GO:0014069 | postsynaptic density | 22 | 118 | 0.0037 |
| CC | GO:0032279 | asymmetric synapse | 22 | 118 | 0.0037 |
| MF | GO:0003779 | actin binding | 43 | 271 | 0.0038 |
| CC | GO:0099513 | polymeric cytoskeletal fiber | 50 | 336 | 0.0039 |
| BP | GO:0050708 | regulation of protein secretion | 23 | 121 | 0.0040 |
| CC | GO:0097060 | synaptic membrane | 28 | 163 | 0.0040 |
| BP | GO:0023051 | regulation of signaling | 232 | 1871 | 0.0041 |
| BP | GO:0032960 | regulation of inositol trisphosphate biosynthetic process | 3 | 4 | 0.0044 |
| BP | GO:0035881 | amacrine cell differentiation | 3 | 4 | 0.0044 |
| BP | GO:0045981 | positive regulation of nucleotide metabolic process | 6 | 16 | 0.0045 |
| BP | GO:1900544 | positive regulation of purine nucleotide metabolic process | 6 | 16 | 0.0045 |
| BP | GO:0044093 | positive regulation of molecular function | 110 | 816 | 0.0045 |
| BP | GO:0016571 | histone methylation | 15 | 68 | 0.0045 |
| BP | GO:0010646 | regulation of cell communication | 231 | 1866 | 0.0045 |
| BP | GO:0003416 | endochondral bone growth | 7 | 21 | 0.0046 |
| CC | GO:0045211 | postsynaptic membrane | 22 | 120 | 0.0046 |
| BP | GO:0021537 | telencephalon development | 27 | 151 | 0.0047 |
| CC | GO:0098688 | parallel fiber to Purkinje cell synapse | 5 | 12 | 0.0047 |
| BP | GO:0006366 | transcription by RNA polymerase II | 118 | 885 | 0.0047 |
| BP | GO:0031323 | regulation of cellular metabolic process | 323 | 2692 | 0.0048 |
| CC | GO:0097431 | mitotic spindle pole | 7 | 22 | 0.0048 |
| CC | GO:0099146 | intrinsic component of postsynaptic density membrane | 7 | 22 | 0.0048 |
| BP | GO:0035556 | intracellular signal transduction | 185 | 1464 | 0.0049 |
| BP | GO:1901019 | regulation of calcium ion transmembrane transporter activity | 9 | 32 | 0.0049 |
| BP | GO:0034968 | histone lysine methylation | 13 | 56 | 0.0050 |
| CC | GO:0005634 | nucleus | 487 | 4363 | 0.0052 |
| BP | GO:0097306 | cellular response to alcohol | 10 | 38 | 0.0052 |
| BP | GO:0006874 | cellular calcium ion homeostasis | 26 | 145 | 0.0052 |
| CC | GO:0045202 | synapse | 86 | 645 | 0.0053 |
| MF | GO:0048027 | mRNA 5'-UTR binding | 5 | 12 | 0.0053 |
| CC | GO:0001527 | microfibril | 4 | 8 | 0.0054 |
| CC | GO:0098839 | postsynaptic density membrane | 8 | 28 | 0.0055 |
| BP | GO:0031326 | regulation of cellular biosynthetic process | 224 | 1812 | 0.0056 |
| CC | GO:0098794 | postsynapse | 43 | 285 | 0.0056 |
| BP | GO:0006817 | phosphate ion transport | 5 | 12 | 0.0056 |
| BP | GO:0023061 | signal release | 39 | 243 | 0.0056 |
| BP | GO:0009892 | negative regulation of metabolic process | 183 | 1452 | 0.0057 |
| BP | GO:0006470 | protein dephosphorylation | 17 | 83 | 0.0058 |
| MF | GO:0008569 | minus-end-directed microtubule motor activity | 6 | 17 | 0.0059 |
| MF | GO:0019789 | SUMO transferase activity | 4 | 8 | 0.0060 |
| MF | GO:0032454 | histone H3-methyl-lysine-9 demethylase activity | 4 | 8 | 0.0060 |
| MF | GO:0051393 | alpha-actinin binding | 4 | 8 | 0.0060 |
| BP | GO:0055074 | calcium ion homeostasis | 27 | 154 | 0.0061 |
| BP | GO:0032502 | developmental process | 414 | 3533 | 0.0062 |
| BP | GO:0051246 | regulation of protein metabolic process | 160 | 1254 | 0.0062 |
| BP | GO:0048715 | negative regulation of oligodendrocyte differentiation | 4 | 8 | 0.0063 |
| BP | GO:0060159 | regulation of dopamine receptor signaling pathway | 4 | 8 | 0.0063 |
| MF | GO:0005515 | protein binding | 482 | 4207 | 0.0064 |
| CC | GO:0043005 | neuron projection | 68 | 496 | 0.0067 |
| BP | GO:0048856 | anatomical structure development | 388 | 3300 | 0.0069 |
| CC | GO:0005856 | cytoskeleton | 166 | 1364 | 0.0070 |
| BP | GO:0043647 | inositol phosphate metabolic process | 8 | 28 | 0.0071 |
| CC | GO:0005886 | plasma membrane | 334 | 2925 | 0.0074 |
| BP | GO:0065009 | regulation of molecular function | 167 | 1321 | 0.0074 |
| BP | GO:0030036 | actin cytoskeleton organization | 60 | 414 | 0.0076 |
| BP | GO:0009306 | protein secretion | 28 | 164 | 0.0077 |
| BP | GO:0010556 | regulation of macromolecule biosynthetic process | 213 | 1727 | 0.0077 |
| MF | GO:0003774 | cytoskeletal motor activity | 12 | 53 | 0.0077 |
| BP | GO:0007420 | brain development | 54 | 366 | 0.0078 |
| CC | GO:0031981 | nuclear lumen | 312 | 2721 | 0.0079 |
| BP | GO:0009954 | proximal/distal pattern formation | 7 | 23 | 0.0080 |
| BP | GO:0098868 | bone growth | 7 | 23 | 0.0080 |
| BP | GO:1902930 | regulation of alcohol biosynthetic process | 7 | 23 | 0.0080 |
| BP | GO:1903532 | positive regulation of secretion by cell | 23 | 128 | 0.0081 |
| BP | GO:0051336 | regulation of hydrolase activity | 74 | 530 | 0.0082 |
| BP | GO:0035592 | establishment of protein localization to extracellular region | 28 | 165 | 0.0083 |
| BP | GO:0021819 | layer formation in cerebral cortex | 5 | 13 | 0.0083 |
| BP | GO:0055012 | ventricular cardiac muscle cell differentiation | 5 | 13 | 0.0083 |
| BP | GO:0097320 | plasma membrane tubulation | 5 | 13 | 0.0083 |
| MF | GO:0003690 | double-stranded DNA binding | 95 | 714 | 0.0084 |
| BP | GO:0019827 | stem cell population maintenance | 19 | 100 | 0.0084 |
| BP | GO:0007154 | cell communication | 384 | 3274 | 0.0085 |
| BP | GO:0030029 | actin filament-based process | 65 | 457 | 0.0085 |
| BP | GO:0001702 | gastrulation with mouth forming second | 6 | 18 | 0.0086 |
| BP | GO:1904894 | positive regulation of receptor signaling pathway via STAT | 6 | 18 | 0.0086 |
| BP | GO:2000036 | regulation of stem cell population maintenance | 6 | 18 | 0.0086 |
| CC | GO:0099572 | postsynaptic specialization | 23 | 134 | 0.0086 |
| CC | GO:0098831 | presynaptic active zone cytoplasmic component | 3 | 5 | 0.0090 |
| BP | GO:0035773 | insulin secretion involved in cellular response to glucose stimulus | 9 | 35 | 0.0092 |
| BP | GO:0098771 | inorganic ion homeostasis | 46 | 306 | 0.0096 |
| MF | GO:0032407 | MutSalpha complex binding | 3 | 5 | 0.0098 |
| MF | GO:0042301 | phosphate ion binding | 3 | 5 | 0.0098 |
| BP | GO:0051254 | positive regulation of RNA metabolic process | 101 | 761 | 0.0099 |
| MF | GO:0004115 | 3',5'-cyclic-AMP phosphodiesterase activity | 4 | 9 | 0.0099 |
| MF | GO:0015278 | calcium-release channel activity | 4 | 9 | 0.0099 |
| BP | GO:0030072 | peptide hormone secretion | 22 | 123 | 0.0100 |
| BP | GO:0060322 | head development | 57 | 395 | 0.0100 |

**Table S12** Analysis of skin transcriptome sequencing data from Plateau Tibetan sheep and Guinan black fur sheep.

| **Sample** | **Reads** | **Bases (G)** | **N (%)** | **Q20 (%)** | **Q30 (%)** |
| --- | --- | --- | --- | --- | --- |
| GB1 | 44688014 | 6.75 | 0.0024 | 97.82 | 96.01 |
| GB2 | 50523652 | 7.63 | 0.0024 | 97.93 | 96.19 |
| GB4 | 52245574 | 7.89 | 0.0024 | 98.06 | 96.39 |
| PT1 | 53978546 | 8.15 | 0.0024 | 98.11 | 96.49 |
| PT2 | 56034534 | 8.46 | 0.0024 | 97.99 | 96.26 |
| PT3 | 52267666 | 7.89 | 0.0024 | 98.03 | 96.35 |
| Average | 51622998 | 7.80 | 0.0024 | 97.99 | 96.28 |

**Table S13** Analysis and comparison of transcriptome data from Plateau Tibetan sheep and Guinan black fur sheep.

| **Sample** | **Clean Reads** | **Clean Data (G)** | **Clean Reads (%)** | **Clean Data (%)** | **Mapped** |
| --- | --- | --- | --- | --- | --- |
| GB1 | 43635278 | 6.57 | 97.64 | 97.30 | 42972433 (98.48%) |
| GB2 | 49506282 | 7.45 | 97.99 | 97.63 | 48762979 (98.50%) |
| GB3 | 51321872 | 7.73 | 98.23 | 97.97 | 50620716 (98.63%) |
| PT1 | 53186258 | 8.01 | 98.53 | 98.27 | 52587069 (98.87%) |
| PT2 | 54959382 | 8.27 | 98.08 | 97.73 | 54278020 (98.76%) |
| PT3 | 51330650 | 7.73 | 98.21 | 97.90 | 50684430 (98.74%) |
| Average | 50656620 | 7.63 | 98.11 | 97.80 | 49984275 (98.66%) |

**Table S14** KEGG enrichment analysis of transcriptome DEGs (*P <* 0.05).

| **PathwayID** | **Pathway** | **DEG_number** | **total_number** | ***P-*value** | **FDR** |
| --- | --- | --- | --- | --- | --- |
| oas04145 | Phagosome | 30 | 163 | 0.0000 | 0.0000 |
| oas04514 | Cell adhesion molecules | 26 | 152 | 0.0000 | 0.0000 |
| oas04650 | Natural killer cell mediated cytotoxicity | 26 | 154 | 0.0000 | 0.0000 |
| oas04612 | Antigen processing and presentation | 17 | 81 | 0.0000 | 0.0000 |
| oas04061 | Viral protein interaction with cytokine and cytokine receptor | 18 | 95 | 0.0000 | 0.0001 |
| oas04060 | Cytokine-cytokine receptor interaction | 35 | 330 | 0.0000 | 0.0008 |
| oas04062 | Chemokine signaling pathway | 23 | 191 | 0.0001 | 0.0019 |
| oas04610 | Complement and coagulation cascades | 13 | 86 | 0.0004 | 0.0053 |
| oas00590 | Arachidonic acid metabolism | 12 | 83 | 0.0010 | 0.0117 |
| oas04620 | Toll-like receptor signaling pathway | 14 | 112 | 0.0017 | 0.0182 |
| oas04672 | Intestinal immune network for IgA production | 8 | 47 | 0.0024 | 0.0252 |
| oas04660 | T cell receptor signaling pathway | 12 | 105 | 0.0073 | 0.0645 |
| oas05340 | Primary immunodeficiency | 6 | 37 | 0.0106 | 0.0905 |
| oas04110 | Cell cycle | 13 | 124 | 0.0108 | 0.0905 |
| oas04670 | Leukocyte transendothelial migration | 12 | 115 | 0.0146 | 0.1073 |
| oas00290 | Valine, leucine and isoleucine biosynthesis | 2 | 4 | 0.0147 | 0.1073 |
| oas04014 | Ras signaling pathway | 20 | 232 | 0.0156 | 0.1085 |
| oas04658 | Th1 and Th2 cell differentiation | 10 | 91 | 0.0177 | 0.1180 |
| oas04659 | Th17 cell differentiation | 11 | 109 | 0.0236 | 0.1500 |
| oas05218 | Melanoma | 8 | 73 | 0.0326 | 0.1862 |
| oas05202 | Transcriptional misregulation in cancer | 16 | 189 | 0.0332 | 0.1862 |
| oas04916 | Melanogenesis | 10 | 101 | 0.0338 | 0.1862 |
| oas05205 | Proteoglycans in cancer | 17 | 207 | 0.0369 | 0.1993 |
| oas04978 | Mineral absorption | 7 | 63 | 0.0414 | 0.2197 |
| oas05143 | African trypanosomiasis | 5 | 38 | 0.0432 | 0.2250 |
| oas00480 | Glutathione metabolism | 7 | 64 | 0.0445 | 0.2257 |
| oas05146 | Amoebiasis | 10 | 106 | 0.0448 | 0.2257 |

**Table S15** GO enrichment analysis of transcriptome DEGs.

| **Category** | **GO.ID** | **Term** | **DEG** | **Total** | ***P-*value** | **FDR** |
| --- | --- | --- | --- | --- | --- | --- |
| BP | GO:0002376 | immune system process | 140 | 1363 | 0.0000 | 0.0000 |
| BP | GO:0006955 | immune response | 90 | 722 | 0.0000 | 0.0000 |
| BP | GO:0002684 | positive regulation of immune system process | 66 | 487 | 0.0000 | 0.0000 |
| CC | GO:0005882 | intermediate filament | 24 | 80 | 0.0000 | 0.0000 |
| BP | GO:0002682 | regulation of immune system process | 87 | 748 | 0.0000 | 0.0000 |
| BP | GO:0045321 | leukocyte activation | 62 | 506 | 0.0000 | 0.0000 |
| BP | GO:0001775 | cell activation | 66 | 566 | 0.0000 | 0.0000 |
| BP | GO:0048856 | anatomical structure development | 245 | 3309 | 0.0000 | 0.0000 |
| CC | GO:0045111 | intermediate filament cytoskeleton | 25 | 115 | 0.0000 | 0.0000 |
| BP | GO:0007275 | multicellular organism development | 225 | 2979 | 0.0000 | 0.0000 |
| BP | GO:0032502 | developmental process | 259 | 3566 | 0.0000 | 0.0000 |
| BP | GO:0032501 | multicellular organismal process | 287 | 4064 | 0.0000 | 0.0000 |
| BP | GO:0050778 | positive regulation of immune response | 40 | 276 | 0.0000 | 0.0000 |
| BP | GO:0048731 | system development | 207 | 2730 | 0.0000 | 0.0000 |
| BP | GO:0048513 | animal organ development | 161 | 1986 | 0.0000 | 0.0000 |
| CC | GO:0005576 | extracellular region | 113 | 1222 | 0.0000 | 0.0000 |
| BP | GO:0006952 | defense response | 78 | 762 | 0.0000 | 0.0000 |
| BP | GO:0050896 | response to stimulus | 320 | 4717 | 0.0000 | 0.0000 |
| BP | GO:0046649 | lymphocyte activation | 51 | 421 | 0.0000 | 0.0000 |
| BP | GO:0098609 | cell-cell adhesion | 51 | 427 | 0.0000 | 0.0000 |
| BP | GO:0006935 | chemotaxis | 42 | 319 | 0.0000 | 0.0000 |
| BP | GO:0042330 | taxis | 42 | 322 | 0.0000 | 0.0000 |
| BP | GO:0002252 | immune effector process | 52 | 445 | 0.0000 | 0.0000 |
| BP | GO:0051239 | regulation of multicellular organismal process | 150 | 1868 | 0.0000 | 0.0000 |
| CC | GO:0099512 | supramolecular fiber | 54 | 461 | 0.0000 | 0.0000 |
| CC | GO:0099081 | supramolecular polymer | 54 | 466 | 0.0000 | 0.0000 |
| BP | GO:0008283 | cell population proliferation | 99 | 1100 | 0.0000 | 0.0000 |
| BP | GO:0006928 | movement of cell or subcellular component | 106 | 1208 | 0.0000 | 0.0001 |
| BP | GO:0002521 | leukocyte differentiation | 43 | 345 | 0.0000 | 0.0001 |
| BP | GO:0042127 | regulation of cell population proliferation | 85 | 904 | 0.0000 | 0.0001 |
| BP | GO:0060326 | cell chemotaxis | 26 | 159 | 0.0000 | 0.0001 |
| BP | GO:0048584 | positive regulation of response to stimulus | 105 | 1206 | 0.0000 | 0.0001 |
| BP | GO:0002520 | immune system development | 62 | 595 | 0.0000 | 0.0001 |
| CC | GO:0045095 | keratin filament | 11 | 31 | 0.0000 | 0.0000 |
| BP | GO:0040011 | locomotion | 95 | 1061 | 0.0000 | 0.0001 |
| BP | GO:0030595 | leukocyte chemotaxis | 22 | 122 | 0.0000 | 0.0001 |
| BP | GO:0048534 | hematopoietic or lymphoid organ development | 59 | 561 | 0.0000 | 0.0001 |
| CC | GO:0099513 | polymeric cytoskeletal fiber | 42 | 336 | 0.0000 | 0.0000 |
| BP | GO:0016477 | cell migration | 80 | 852 | 0.0000 | 0.0001 |
| BP | GO:0022610 | biological adhesion | 72 | 746 | 0.0000 | 0.0002 |
| BP | GO:0002694 | regulation of leukocyte activation | 38 | 301 | 0.0000 | 0.0002 |
| BP | GO:0001942 | hair follicle development | 15 | 64 | 0.0000 | 0.0002 |
| BP | GO:0008284 | positive regulation of cell population proliferation | 54 | 507 | 0.0000 | 0.0002 |
| MF | GO:0000981 | DNA-binding transcription factor activity, RNA polymerase II-specific | 54 | 508 | 0.0000 | 0.0013 |
| BP | GO:0007155 | cell adhesion | 71 | 740 | 0.0000 | 0.0002 |
| BP | GO:0050731 | positive regulation of peptidyl-tyrosine phosphorylation | 19 | 100 | 0.0000 | 0.0002 |
| BP | GO:0042100 | B cell proliferation | 14 | 57 | 0.0000 | 0.0002 |
| BP | GO:0051249 | regulation of lymphocyte activation | 33 | 248 | 0.0000 | 0.0002 |
| BP | GO:0050776 | regulation of immune response | 44 | 382 | 0.0000 | 0.0002 |
| BP | GO:0003002 | regionalization | 31 | 227 | 0.0000 | 0.0002 |
| BP | GO:0018108 | peptidyl-tyrosine phosphorylation | 24 | 152 | 0.0000 | 0.0003 |
| BP | GO:0009617 | response to bacterium | 37 | 299 | 0.0000 | 0.0003 |
| BP | GO:0022404 | molting cycle process | 15 | 67 | 0.0000 | 0.0003 |
| BP | GO:0022405 | hair cycle process | 15 | 67 | 0.0000 | 0.0003 |
| BP | GO:0098773 | skin epidermis development | 15 | 67 | 0.0000 | 0.0003 |
| BP | GO:0042303 | molting cycle | 16 | 76 | 0.0000 | 0.0003 |
| BP | GO:0042633 | hair cycle | 16 | 76 | 0.0000 | 0.0003 |
| BP | GO:0030888 | regulation of B cell proliferation | 12 | 44 | 0.0000 | 0.0003 |
| BP | GO:0070663 | regulation of leukocyte proliferation | 22 | 133 | 0.0000 | 0.0003 |
| BP | GO:0018212 | peptidyl-tyrosine modification | 24 | 154 | 0.0000 | 0.0003 |
| BP | GO:0007389 | pattern specification process | 35 | 278 | 0.0000 | 0.0003 |
| BP | GO:0032103 | positive regulation of response to external stimulus | 30 | 221 | 0.0000 | 0.0003 |
| BP | GO:0002688 | regulation of leukocyte chemotaxis | 15 | 69 | 0.0000 | 0.0003 |
| BP | GO:0042110 | T cell activation | 35 | 281 | 0.0000 | 0.0004 |
| BP | GO:0050670 | regulation of lymphocyte proliferation | 21 | 126 | 0.0000 | 0.0004 |
| MF | GO:0030545 | receptor regulator activity | 37 | 307 | 0.0000 | 0.0019 |
| BP | GO:0009605 | response to external stimulus | 111 | 1359 | 0.0000 | 0.0004 |
| BP | GO:0048870 | cell motility | 84 | 953 | 0.0000 | 0.0004 |
| BP | GO:0051674 | localization of cell | 84 | 953 | 0.0000 | 0.0004 |
| BP | GO:0048869 | cellular developmental process | 171 | 2324 | 0.0000 | 0.0004 |
| BP | GO:0030154 | cell differentiation | 168 | 2275 | 0.0000 | 0.0004 |
| BP | GO:0048598 | embryonic morphogenesis | 44 | 397 | 0.0000 | 0.0004 |
| BP | GO:2000026 | regulation of multicellular organismal development | 100 | 1194 | 0.0000 | 0.0004 |
| BP | GO:0032944 | regulation of mononuclear cell proliferation | 21 | 128 | 0.0000 | 0.0004 |
| BP | GO:0030097 | hemopoiesis | 54 | 530 | 0.0000 | 0.0004 |
| BP | GO:0050865 | regulation of cell activation | 38 | 323 | 0.0000 | 0.0004 |
| CC | GO:0099080 | supramolecular complex | 63 | 644 | 0.0000 | 0.0004 |
| BP | GO:0050730 | regulation of peptidyl-tyrosine phosphorylation | 22 | 141 | 0.0000 | 0.0006 |
| BP | GO:0002009 | morphogenesis of an epithelium | 39 | 340 | 0.0000 | 0.0006 |
| BP | GO:0048729 | tissue morphogenesis | 44 | 404 | 0.0000 | 0.0006 |
| BP | GO:0046651 | lymphocyte proliferation | 24 | 163 | 0.0000 | 0.0006 |
| BP | GO:0050789 | regulation of biological process | 395 | 6357 | 0.0000 | 0.0006 |
| BP | GO:0009952 | anterior/posterior pattern specification | 22 | 142 | 0.0000 | 0.0006 |
| BP | GO:1902105 | regulation of leukocyte differentiation | 24 | 164 | 0.0000 | 0.0006 |
| BP | GO:0070661 | leukocyte proliferation | 25 | 175 | 0.0000 | 0.0006 |
| BP | GO:0009790 | embryo development | 67 | 721 | 0.0000 | 0.0006 |
| BP | GO:0048863 | stem cell differentiation | 20 | 122 | 0.0000 | 0.0006 |
| BP | GO:0002683 | negative regulation of immune system process | 32 | 256 | 0.0000 | 0.0006 |
| BP | GO:0032943 | mononuclear cell proliferation | 24 | 165 | 0.0000 | 0.0007 |
| BP | GO:0060429 | epithelium development | 66 | 709 | 0.0000 | 0.0007 |
| BP | GO:0002761 | regulation of myeloid leukocyte differentiation | 14 | 66 | 0.0000 | 0.0007 |
| BP | GO:0006959 | humoral immune response | 16 | 84 | 0.0000 | 0.0007 |
| BP | GO:0045595 | regulation of cell differentiation | 88 | 1033 | 0.0000 | 0.0007 |
| BP | GO:0006956 | complement activation | 9 | 28 | 0.0000 | 0.0007 |
| BP | GO:0009653 | anatomical structure morphogenesis | 126 | 1624 | 0.0000 | 0.0007 |
| BP | GO:0048732 | gland development | 31 | 248 | 0.0000 | 0.0008 |
| BP | GO:0009887 | animal organ morphogenesis | 60 | 630 | 0.0000 | 0.0008 |
| BP | GO:0050793 | regulation of developmental process | 120 | 1534 | 0.0000 | 0.0008 |
| BP | GO:0014033 | neural crest cell differentiation | 12 | 51 | 0.0000 | 0.0009 |
| BP | GO:0030879 | mammary gland development | 15 | 77 | 0.0000 | 0.0009 |
| MF | GO:0048018 | receptor ligand activity | 34 | 287 | 0.0000 | 0.0041 |
| BP | GO:0030098 | lymphocyte differentiation | 30 | 239 | 0.0000 | 0.0009 |
| BP | GO:0002366 | leukocyte activation involved in immune response | 23 | 159 | 0.0000 | 0.0009 |
| BP | GO:0051240 | positive regulation of multicellular organismal process | 88 | 1045 | 0.0000 | 0.0010 |
| BP | GO:0097529 | myeloid leukocyte migration | 18 | 108 | 0.0000 | 0.0011 |
| BP | GO:0002263 | cell activation involved in immune response | 23 | 161 | 0.0000 | 0.0011 |
| BP | GO:0050900 | leukocyte migration | 27 | 207 | 0.0000 | 0.0012 |
| BP | GO:0051094 | positive regulation of developmental process | 71 | 799 | 0.0000 | 0.0012 |
| MF | GO:0003700 | DNA-binding transcription factor activity | 59 | 627 | 0.0000 | 0.0041 |
| BP | GO:0045637 | regulation of myeloid cell differentiation | 19 | 119 | 0.0000 | 0.0012 |
| BP | GO:0048562 | embryonic organ morphogenesis | 26 | 196 | 0.0000 | 0.0012 |
| MF | GO:0030546 | signaling receptor activator activity | 34 | 293 | 0.0000 | 0.0041 |
| BP | GO:0007059 | chromosome segregation | 27 | 208 | 0.0000 | 0.0012 |
| BP | GO:0042476 | odontogenesis | 15 | 80 | 0.0000 | 0.0012 |
| BP | GO:0007166 | cell surface receptor signaling pathway | 113 | 1444 | 0.0000 | 0.0013 |
| MF | GO:0042379 | chemokine receptor binding | 10 | 38 | 0.0000 | 0.0041 |
| BP | GO:0002690 | positive regulation of leukocyte chemotaxis | 12 | 54 | 0.0000 | 0.0014 |
| CC | GO:0009897 | external side of plasma membrane | 22 | 150 | 0.0000 | 0.0015 |
| BP | GO:0065007 | biological regulation | 415 | 6807 | 0.0000 | 0.0015 |
| BP | GO:0048762 | mesenchymal cell differentiation | 20 | 132 | 0.0000 | 0.0015 |
| BP | GO:0042063 | gliogenesis | 23 | 165 | 0.0000 | 0.0015 |
| BP | GO:0070665 | positive regulation of leukocyte proliferation | 15 | 82 | 0.0000 | 0.0016 |
| BP | GO:0061180 | mammary gland epithelium development | 11 | 47 | 0.0000 | 0.0017 |
| BP | GO:0002443 | leukocyte mediated immunity | 25 | 190 | 0.0000 | 0.0018 |
| BP | GO:0050864 | regulation of B cell activation | 15 | 83 | 0.0000 | 0.0018 |
| BP | GO:0007165 | signal transduction | 201 | 2916 | 0.0000 | 0.0019 |
| BP | GO:0002573 | myeloid leukocyte differentiation | 20 | 135 | 0.0000 | 0.0020 |
| BP | GO:0050920 | regulation of chemotaxis | 18 | 114 | 0.0000 | 0.0020 |
| BP | GO:0048864 | stem cell development | 11 | 48 | 0.0000 | 0.0020 |
| BP | GO:0030183 | B cell differentiation | 15 | 84 | 0.0000 | 0.0020 |
| BP | GO:0009888 | tissue development | 93 | 1149 | 0.0000 | 0.0020 |
| MF | GO:0004668 | protein-arginine deiminase activity | 4 | 5 | 0.0000 | 0.0059 |
| BP | GO:0018101 | protein citrullination | 4 | 5 | 0.0000 | 0.0020 |
| BP | GO:0051241 | negative regulation of multicellular organismal process | 64 | 715 | 0.0000 | 0.0021 |
| BP | GO:1903706 | regulation of hemopoiesis | 28 | 229 | 0.0000 | 0.0022 |
| BP | GO:0002763 | positive regulation of myeloid leukocyte differentiation | 9 | 33 | 0.0000 | 0.0022 |
| BP | GO:0001755 | neural crest cell migration | 8 | 26 | 0.0000 | 0.0023 |
| BP | GO:0007154 | cell communication | 219 | 3241 | 0.0000 | 0.0024 |
| BP | GO:0009607 | response to biotic stimulus | 61 | 676 | 0.0001 | 0.0024 |
| BP | GO:0048706 | embryonic skeletal system development | 16 | 96 | 0.0001 | 0.0025 |
| CC | GO:0098552 | side of membrane | 29 | 238 | 0.0001 | 0.0029 |
| BP | GO:0098742 | cell-cell adhesion via plasma-membrane adhesion molecules | 18 | 117 | 0.0001 | 0.0026 |
| BP | GO:0043588 | skin development | 22 | 161 | 0.0001 | 0.0026 |
| BP | GO:0060485 | mesenchyme development | 22 | 161 | 0.0001 | 0.0026 |
| BP | GO:0002253 | activation of immune response | 21 | 150 | 0.0001 | 0.0026 |
| BP | GO:0050921 | positive regulation of chemotaxis | 14 | 77 | 0.0001 | 0.0026 |
| BP | GO:0051783 | regulation of nuclear division | 17 | 107 | 0.0001 | 0.0026 |
| BP | GO:2000106 | regulation of leukocyte apoptotic process | 11 | 50 | 0.0001 | 0.0026 |
| CC | GO:0009986 | cell surface | 41 | 391 | 0.0001 | 0.0030 |
| BP | GO:1904892 | regulation of receptor signaling pathway via STAT | 13 | 68 | 0.0001 | 0.0027 |
| BP | GO:0042475 | odontogenesis of dentin-containing tooth | 12 | 59 | 0.0001 | 0.0027 |
| BP | GO:0042113 | B cell activation | 22 | 163 | 0.0001 | 0.0029 |
| BP | GO:0000070 | mitotic sister chromatid segregation | 16 | 98 | 0.0001 | 0.0029 |
| BP | GO:0060562 | epithelial tube morphogenesis | 27 | 223 | 0.0001 | 0.0031 |
| BP | GO:0042531 | positive regulation of tyrosine phosphorylation of STAT protein | 9 | 35 | 0.0001 | 0.0032 |
| BP | GO:0090025 | regulation of monocyte chemotaxis | 6 | 15 | 0.0001 | 0.0034 |
| BP | GO:0048568 | embryonic organ development | 32 | 287 | 0.0001 | 0.0034 |
| BP | GO:0070371 | ERK1 and ERK2 cascade | 21 | 154 | 0.0001 | 0.0035 |
| BP | GO:0002274 | myeloid leukocyte activation | 18 | 121 | 0.0001 | 0.0036 |
| BP | GO:0042471 | ear morphogenesis | 14 | 80 | 0.0001 | 0.0037 |
| BP | GO:0030099 | myeloid cell differentiation | 29 | 251 | 0.0001 | 0.0038 |
| BP | GO:0014032 | neural crest cell development | 10 | 44 | 0.0001 | 0.0038 |
| BP | GO:1904894 | positive regulation of receptor signaling pathway via STAT | 10 | 44 | 0.0001 | 0.0038 |
| BP | GO:0043299 | leukocyte degranulation | 9 | 36 | 0.0001 | 0.0038 |
| BP | GO:0051707 | response to other organism | 58 | 649 | 0.0001 | 0.0041 |
| MF | GO:0004175 | endopeptidase activity | 35 | 330 | 0.0001 | 0.0132 |
| BP | GO:0051445 | regulation of meiotic cell cycle | 8 | 29 | 0.0001 | 0.0043 |
| BP | GO:0071675 | regulation of mononuclear cell migration | 8 | 29 | 0.0001 | 0.0043 |
| BP | GO:0043207 | response to external biotic stimulus | 58 | 651 | 0.0001 | 0.0043 |
| CC | GO:0000775 | chromosome, centromeric region | 18 | 121 | 0.0001 | 0.0051 |
| BP | GO:0098542 | defense response to other organism | 44 | 453 | 0.0001 | 0.0045 |
| BP | GO:0006954 | inflammatory response | 37 | 358 | 0.0001 | 0.0045 |
| BP | GO:0007186 | G protein-coupled receptor signaling pathway | 31 | 280 | 0.0001 | 0.0046 |
| BP | GO:0050808 | synapse organization | 26 | 218 | 0.0001 | 0.0048 |
| BP | GO:0008544 | epidermis development | 22 | 170 | 0.0001 | 0.0048 |
| BP | GO:0001501 | skeletal system development | 35 | 334 | 0.0001 | 0.0051 |
| BP | GO:0023052 | signaling | 214 | 3203 | 0.0001 | 0.0051 |
| BP | GO:0030001 | metal ion transport | 36 | 348 | 0.0001 | 0.0052 |
| BP | GO:2000107 | negative regulation of leukocyte apoptotic process | 8 | 30 | 0.0001 | 0.0053 |
| BP | GO:0002685 | regulation of leukocyte migration | 17 | 115 | 0.0001 | 0.0053 |
| BP | GO:0002250 | adaptive immune response | 24 | 196 | 0.0002 | 0.0054 |
| BP | GO:0002455 | humoral immune response mediated by circulating immunoglobulin | 7 | 23 | 0.0002 | 0.0054 |
| BP | GO:0009954 | proximal/distal pattern formation | 7 | 23 | 0.0002 | 0.0054 |
| BP | GO:0040020 | regulation of meiotic nuclear division | 7 | 23 | 0.0002 | 0.0054 |
| CC | GO:0005615 | extracellular space | 55 | 601 | 0.0002 | 0.0064 |
| BP | GO:0033141 | positive regulation of peptidyl-serine phosphorylation of STAT protein | 3 | 3 | 0.0002 | 0.0055 |
| BP | GO:0098883 | synapse pruning | 3 | 3 | 0.0002 | 0.0055 |
| BP | GO:1903613 | regulation of protein tyrosine phosphatase activity | 3 | 3 | 0.0002 | 0.0055 |
| BP | GO:0002699 | positive regulation of immune effector process | 19 | 138 | 0.0002 | 0.0055 |
| BP | GO:0030155 | regulation of cell adhesion | 41 | 418 | 0.0002 | 0.0055 |
| MF | GO:0005102 | signaling receptor binding | 73 | 884 | 0.0002 | 0.0166 |
| MF | GO:0004714 | transmembrane receptor protein tyrosine kinase activity | 14 | 85 | 0.0002 | 0.0166 |
| BP | GO:0022407 | regulation of cell-cell adhesion | 27 | 235 | 0.0002 | 0.0061 |
| MF | GO:0008009 | chemokine activity | 8 | 31 | 0.0002 | 0.0166 |
| BP | GO:0051716 | cellular response to stimulus | 248 | 3818 | 0.0002 | 0.0065 |
| BP | GO:0046425 | regulation of receptor signaling pathway via JAK-STAT | 12 | 66 | 0.0002 | 0.0065 |
| BP | GO:0050863 | regulation of T cell activation | 22 | 175 | 0.0002 | 0.0065 |
| BP | GO:0001934 | positive regulation of protein phosphorylation | 47 | 506 | 0.0002 | 0.0066 |
| MF | GO:0005125 | cytokine activity | 17 | 118 | 0.0002 | 0.0166 |
| BP | GO:0044419 | interspecies interaction between organisms | 65 | 769 | 0.0002 | 0.0066 |
| BP | GO:0050671 | positive regulation of lymphocyte proliferation | 13 | 76 | 0.0002 | 0.0066 |
| BP | GO:1905330 | regulation of morphogenesis of an epithelium | 13 | 76 | 0.0002 | 0.0066 |
| BP | GO:0002275 | myeloid cell activation involved in immune response | 10 | 48 | 0.0002 | 0.0067 |
| BP | GO:0014031 | mesenchymal cell development | 10 | 48 | 0.0002 | 0.0067 |
| BP | GO:0070838 | divalent metal ion transport | 24 | 200 | 0.0002 | 0.0067 |
| BP | GO:0045597 | positive regulation of cell differentiation | 49 | 536 | 0.0002 | 0.0068 |
| MF | GO:0005509 | calcium ion binding | 46 | 494 | 0.0002 | 0.0166 |
| BP | GO:0048518 | positive regulation of biological process | 223 | 3385 | 0.0002 | 0.0071 |
| BP | GO:0032946 | positive regulation of mononuclear cell proliferation | 13 | 77 | 0.0002 | 0.0073 |
| BP | GO:0097696 | receptor signaling pathway via STAT | 13 | 77 | 0.0002 | 0.0073 |
| BP | GO:0030334 | regulation of cell migration | 48 | 524 | 0.0002 | 0.0074 |
| BP | GO:0002695 | negative regulation of leukocyte activation | 15 | 98 | 0.0002 | 0.0075 |
| MF | GO:0003690 | double-stranded DNA binding | 61 | 715 | 0.0002 | 0.0175 |
| BP | GO:0051251 | positive regulation of lymphocyte activation | 20 | 155 | 0.0003 | 0.0082 |
| BP | GO:0030889 | negative regulation of B cell proliferation | 5 | 12 | 0.0003 | 0.0082 |
| BP | GO:0090026 | positive regulation of monocyte chemotaxis | 5 | 12 | 0.0003 | 0.0082 |
| MF | GO:1990837 | sequence-specific double-stranded DNA binding | 58 | 674 | 0.0003 | 0.0187 |
| BP | GO:0072511 | divalent inorganic cation transport | 24 | 204 | 0.0003 | 0.0086 |
| BP | GO:0010562 | positive regulation of phosphorus metabolic process | 51 | 572 | 0.0003 | 0.0087 |
| BP | GO:0045937 | positive regulation of phosphate metabolic process | 51 | 572 | 0.0003 | 0.0087 |
| BP | GO:0002696 | positive regulation of leukocyte activation | 22 | 180 | 0.0003 | 0.0088 |
| BP | GO:0030217 | T cell differentiation | 21 | 168 | 0.0003 | 0.0088 |
| BP | GO:0002224 | toll-like receptor signaling pathway | 13 | 79 | 0.0003 | 0.0089 |

**Table S16** Genes that are upregulated and downregulated in the melanogenesis pathway.

| **Genes** | **Up-regulated** | **Down-regulated** |
| --- | --- | --- |
|  | *KIT* | *FZD2* |
|  | *TYRP1* | *LEF1* |
|  | *TYR* | *FZD3* |
|  | *MC1R* |  |
|  | *WNT4* |  |
|  | *PLCB2* |  |

**Table S17** qPCR Validation of RNA-Seq Data

| **Gene** | **RNA-seq: log_₂_(Fold Change)** | **qPCR: log_₂_(Fold Change)** | ***r*** | ***P*-value** |
| --- | --- | --- | --- | --- |
| *PLCB2* | 1.27 | 1.07 | 0.92 | ***P*<0.0001** |
| *WNT4* | 1.69 | -0.06 |  |  |
| *CCR3* | 1.94 | 0.64 |  |  |
| *FZD2* | -1.25 | -1.27 |  |  |
| *FZD3* | -1.00 | -1.31 |  |  |
| *MC1R* | 2.26 | -0.53 |  |  |
| *TYR* | 6.92 | 1.30 |  |  |
| *KIT* | 2.03 | 1.15 |  |  |
| *LEF1* | -1.24 | -1.27 |  |  |
| *TYRP1* | 11.33 | 6.07 |  |  |

**Table S18** SNPs information table.

| **SNP** | **Position** | **Type** | **Exon** | ****Sequence (F’—R’)**** |
| --- | --- | --- | --- | --- |
| *KIT*-SNP1 | rs404963453 | Synonymous | 15 | GACTT[G/C]CTGAG |
| *KIT*-SNP2 | rs416169878 | Nonsynonymous (Ala/Val) | 15 | CATGG[C/T]GTTCC |
| *MITF*-SNP1 | g.31615279302 | Synonymous | 4 | AGCGA[A/T]TGCCC |
| *MITF*-SNP2 | rs161248978 | Synonymous | 9 | CACTT[G/A]TTACT |
| *MITF*-SNP3 | rs161248955 | Synonymous | 10 | TCCCC[T/C]GGAGC |
| *MITF*-SNP4 | rs416988818 | Synonymous | 10 | GAAAC[C/T]GAACA |
| *MC1R*-SNP1 | rs3508196008 | Nonsynonymous (Met/Lys) | 1 | CCCCA[T/A]GTACT |
| *MC1R*-SNP2 | rs409651063 | Nonsynonymous (Asp/Asn) | 1 | TCCGG[G/A]ACGTG |
| *MC1R*-SNP3 | rs160910030 | Synonymous | 1 | CGCTA[C/T]ATCTC |
| *MC1R*-SNP4 | rs398814350 | Synonymous | 1 | ATGCT[T/G]GCCCT |
| *MC1R*-SNP5 | rs412064209 | Synonymous | 1 | ACCAT[C/T]CTGCT |
| *MC1R*-SNP6 | rs596064420 | Nonsynonymous (Glu/Pro) | 1 | CTCGC[T/C]CATCG |


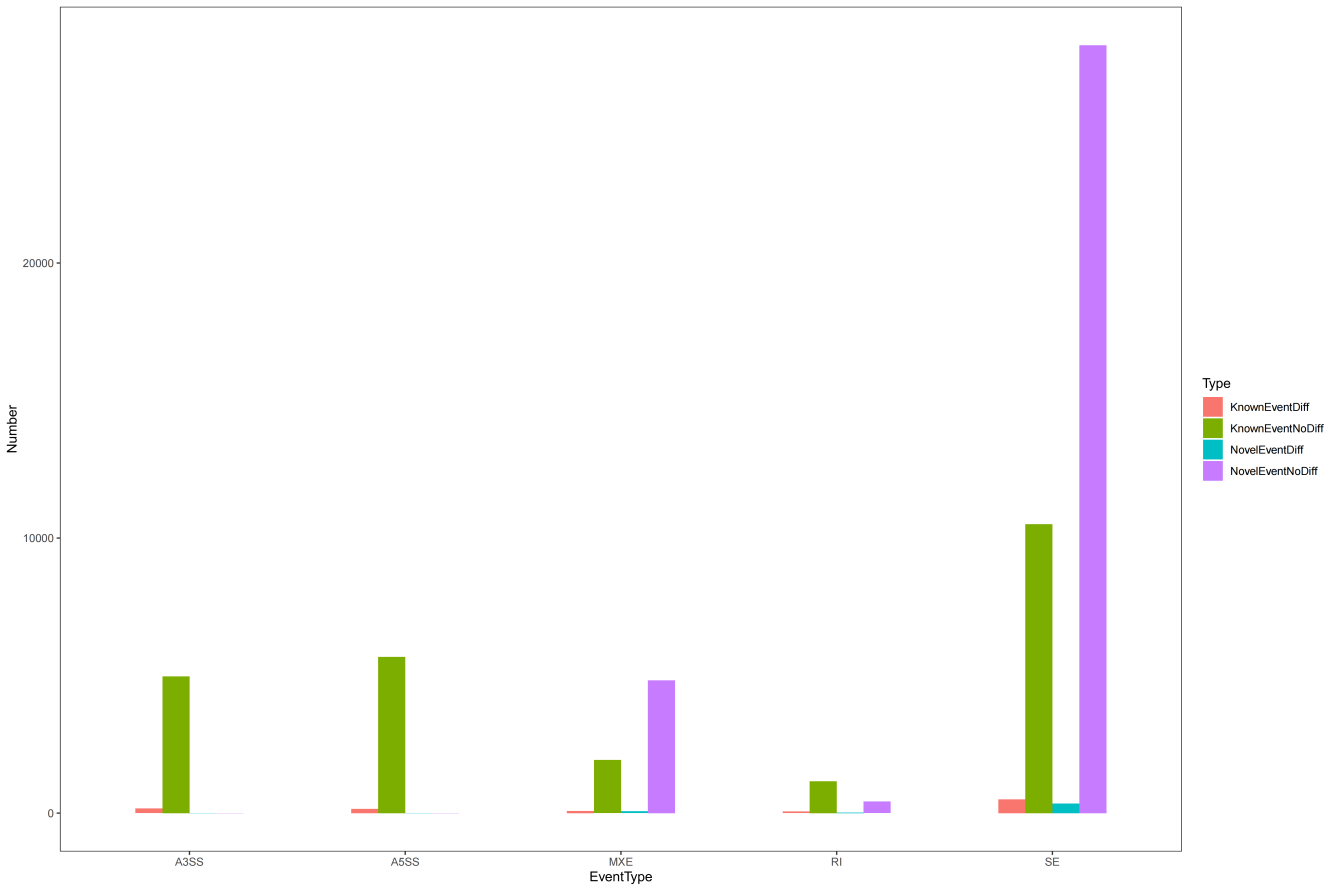


**Figure S1** Alternative splicing analysis. A5SS: alternative 5' splice site usage; MXE: mutually exclusive exons; RI: retained introns; SE: skipped exons.The x-axis displays the five types of alternative splicing events identified by rMATS, while the y-axis represents the number of transcripts. Red indicates known alternative splicing events with significant differential occurrence, green indicates known alternative splicing events without significant differential occurrence, blue indicates novel alternative splicing events with significant differential occurrence, and purple indicates novel alternative splicing events without significant differential occurrence.
